# Tracking Metabolic Responses to Citalopram in Colon Cells with Raman Spectroscopy

**DOI:** 10.1021/acs.analchem.5c03360

**Published:** 2026-02-06

**Authors:** Karolina Beton-Mysur, Beata Brozek-Pluska

**Affiliations:** Faculty of Chemistry, Institute of Applied Radiation Chemistry, Laboratory of Laser Molecular Spectroscopy, 49584 Lodz University of Technology, Wroblewskiego 15, 93-590 Lodz, Poland

## Abstract

The antidepressant citalopram, primarily known for its selective serotonin reuptake inhibitor properties, has attracted interest in the broader scope of its effects on cellular metabolism beyond the brain. This study explores metabolic alterations induced by citalopram in human colon cells, both normal and cancerous. Understanding the fate of citalopram in the colon environment is crucial for assessing its systemic and localized effects, particularly in the context of the colon–brain axis and metabolic reprogramming observed in cancer cells. We review the impact of citalopram on metabolic pathways, its transformation in colon cells, and differences between normal and cancerous cells in handling this compound. This study demonstrates that citalopram induces metabolic alterations in cancerous Caco-2 (G1), LoVo (G4), and normal CCD-18Co colon cells. Using Raman spectroscopy and imaging, distinct biochemical changes were identified in the endoplasmic reticulum and lipid droplets after treatment. These changes were reflected in Raman band intensity ratios 1080/1292 cm^–1^, 1004/1660 cm^–1^, and 1444/1266 cm^–1^. Citalopram treatment led to altered lipid ratios (I_1444/1266_) and increased nucleic acid (I_1080/1292_) and protein (I_1004/1660_) ratios, especially in cancerous cells, suggesting modulated lipogenesis, altered transcriptional activity, and endoplasmic reticulum stress-related protein changes. Raman spectroscopy proved to be a label-free method for monitoring drug-induced metabolic responses at the subcellular level. Citalopram also significantly reduced endoplasmic reticulum and lipid droplet areas in Caco-2 and LoVo cells, indicating disrupted cellular homeostasis and increased sensitivity to toxin-induced stress. In contrast, normal CCD-18Co fibroblasts showed increased lipid droplet accumulation, suggesting an adaptive detoxification response.

## Graphical abstract

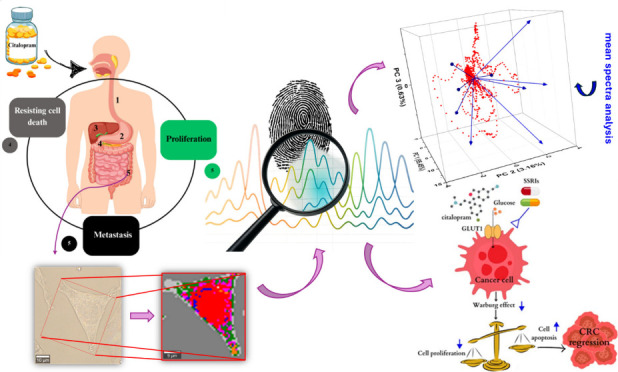

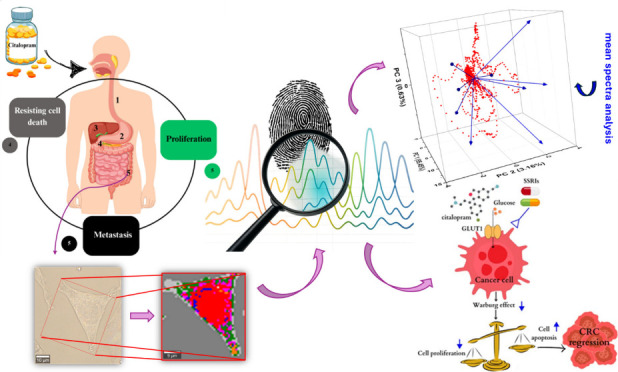

## Introduction

Colorectal cancer (CRC) is one of the most prevalent malignancies worldwide, ranking as the third most commonly diagnosed cancer and the second leading cause of cancer-related mortality. According to the Global Cancer Observatory (GLOBOCAN), in 2020 alone, approximately 1.9 million new cases of CRC were diagnosed, and over 900,000 deaths were attributed to the disease. Despite advancements in early detection and treatment strategies, the prognosis for metastatic CRC remains poor, necessitating the exploration of novel therapeutic approaches. Emerging evidence suggests that targeting cellular metabolism in CRC cells may provide a promising avenue for intervention.^1–4^

The physical impact of CRC and its treatment can be daunting for patients. Compared to the general population, those diagnosed with CRC face significant limitations in physical activities. Physical and functional difficulties often lead to considerable psychological distress, adversely affecting overall health and quality of life. In 2019, Peng et al. found that up to 47.2% of CRC patients experienced anxiety, while depression affected as many as 57%. Additionally, a 2021 cohort study in Denmark showed that CRC patients had a much higher risk of depression compared to those without cancer, even five years into the study.^5^

Citalopram, a widely prescribed selective serotonin reuptake inhibitor (SSRI), is primarily used in the treatment of depression, anxiety disorders, and other mood-related conditions, has gained attention for its potential antineoplastic properties. Several studies indicate that SSRIs, including citalopram, may exert anticancer effects by modulating apoptotic pathways, inhibiting cell proliferation, and affecting cellular metabolism.^6–13^ Although the drug’s effect on neurotransmission has been well studied, the broader pharmacological and metabolic consequences remain less understood, particularly within the gastrointestinal tract.

The human colon is a complex environment with a tightly regulated metabolism to maintain physiological functions. Moreover, the reprogramming of metabolism is a hallmark of cancer cells, including those of the gastrointestinal tract.^14–16^ Therefore, it is crucial to study how normal and cancerous colon cells respond to citalopram supplementation, focusing on metabolic changes that may impact cellular function and therapeutic efficacy.

Figure 1 shows the inhibitory potential of citalopram on carcinogenic processes in CRC, and the side effects of citalopram.

**Figure 1:**
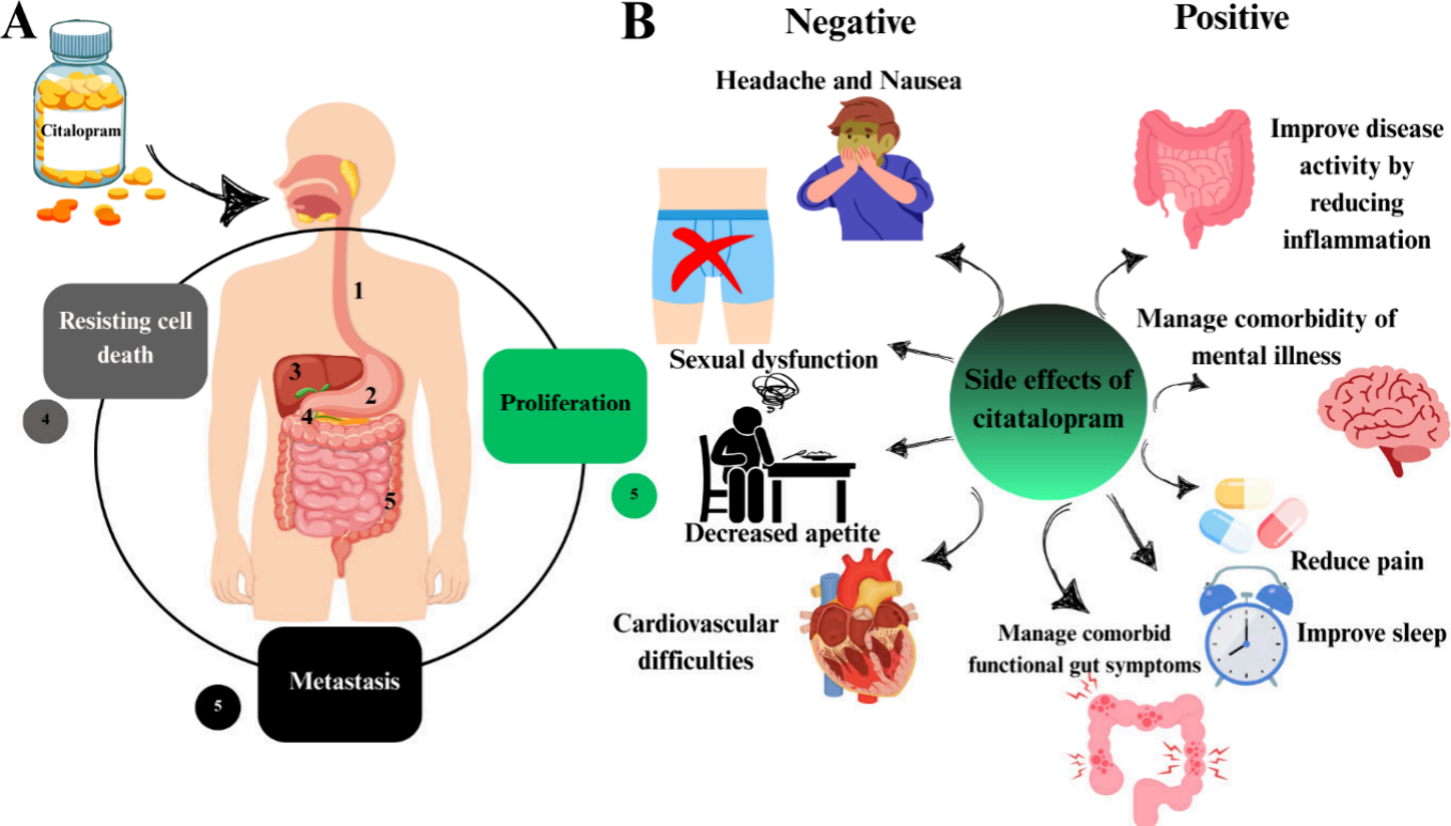
Refocusing psychiatric drugs in gastrointestinal cancers, showing inhibitory potential on carcinogenic processes such as growth, proliferation, angiogenesis, metastasis, and cell survival in colorectal cancer (5) (A) and the side effects of citalopram (B).

In the colon, citalopram is absorbed through the colon epithelial cells, where it may undergo both phase I (oxidation, reduction, and hydrolysis) and phase II (conjugation with glucuronic acid, sulfate) metabolic reactions. Enzymes such as those from the cytochrome P450 (CYP450) family, found in the colon epithelium, may participate in these processes, although their contribution to overall metabolism is typically less significant than the liver’s. Notably, the colon microbiota can also influence the metabolism of citalopram, potentially altering its bioavailability and activity.^17–20^

This study aims to investigate the effects of citalopram on metabolic pathways in gastrointestinal cells, with a particular focus on its potential to alter key metabolic processes. By elucidating the mechanistic underpinnings of citalopram’s impact on normal and cancer cell metabolism and its biotransformation in the colon, this research may pave the way for novel therapeutic strategies that repurpose existing pharmacological agents for metabolic effects and their changes induced by drug therapy.

## Materials and Methods

### Chemical Compounds

Cital (Citalopram) has been purchased commercially from Neuraxpharm Arzneimittel GmbH. Phosphate Buffered Saline (PBS) was purchased from Gibco, catalog number: 10010023 (pH 7.4 at 25 °C, 0.01 M). Cell Proliferation Kit II was purchased from Merck Life Science Sp. z o.o., an affiliate of Merck KGaA, Darmstadt, Germany, acting as a distributor of Roche Diagnostics GmbH, catalog number: 11465015001.

### Cell Culturing

The research subject was CCD-18 Co (ATCC CRL-1459), Caco-2 (ATCC HTB-37), and LoVo (ATCC CCL-229) cell line purchased from ATCC: The Global Bioresource Center. Characteristics and morphology of the CCD-18 Co cells are defined as normal colon myofibroblasts. The CaCo-2 cell line was obtained from a patient diagnosed with colon adenocarcinoma. LoVo is a cell line derived from the large intestine of a grade IV Dukes’ C colorectal cancer patient. CCD-18 Co and Caco-2 cell line was cultured using ATCC-formulated Eagle’s Minimum Essential Medium with l-glutamine (product code ATCC-30–2003). To make the complete growth medium, fetal bovine serum (FBS) was added at final concentrations of 10% and 20%, respectively. LoVo cell line was cultured using ATCC-formulated F-12K Medium (Kaighn’s Modification of Ham’s F-12 Medium) (product code ATCC-30–2004). To make the complete growth medium, FBS was added to a final concentration of 10%. The culture medium was renewed every 2–3 days. The biological safety of both CCD-18 Co, Caco-2 cell, and LoVo cell lines has been classified by the American Biosafety Association (ABSA) as level 1 (BSL-1).

### Cultivation Conditions

Cell lines (CCD-18Co, Caco-2, LoVo) used in the experiments in this study were grown in flat-bottom culture flasks made of plasma-treated polystyrene with a cell culture surface area of 75 cm^2^. Flasks containing cells were stored in an incubator providing environmental conditions at 37 °C, 5% CO_2_, 95% air.

### Cell Treatment

CCD-18 Co, Caco-2, and LoVo cells, seeded onto calcium fluoride (CaF_2_) windows (25 × 1 mm) at a density of 10^3^ cells/cm^3^, were incubated for 24 h. After this time for CCD-18 Co, Caco-2, and LoVo cells, the standard growth medium was removed, and 20 mg of Citalopram solution (in growth medium) was added. The time of incubation was 3 and 24 h. Measurements for pure CCD-18 Co, Caco-2, and LoVo cells have been done after 24 h of incubation in pure growth medium. For Raman measurements, the cells were washed twice with PBS and fixed with 4% formalin solution. All experiments were conducted on freshly fixed cells in the PBS solution.

### Raman Spectra and Imaging Acquisition and Analysis

Raman imaging and spectral measurements were carried out on a confocal Raman microscope (alpha 300 RSA+, WITec, Ulm, Germany) equipped with an Olympus optical system, a high-throughput monochromator (UHTS), and a thermoelectrically cooled EMCCD detector (Newton DU970N-UVB-353, Andor Technology, Belfast, Northern Ireland; 1600 × 200 pixels, 16 μm, operating at -60 °C with vertical binning). A 532 nm laser source was coupled to the system through a 50 μm optical fiber and focused onto the specimens using a 40× water immersion objective (Zeiss W Plan-Apochromat, NA = 1.0, VIS-IR, working distance 2.5 mm). The laser power at the sample plane was maintained at 10 mW. Spectra were collected with a 1200 lines mm^–1^ diffraction grating in two regions: 500–1800 cm^–1^ (integration time 0.5 s) and 2700–3150 cm^–1^ (integration time 0.3 s). Elastic scattering was suppressed with appropriate edge filters, and calibration was routinely performed using the Si reference peak at 520.7 cm^–1^. Raman maps were acquired with a spatial step size of 1 × 1 μm and one spectrum per pixel. The measurements did not require any prior chemical or physical preparation of the biological material. For all experimental variants, Raman spectroscopy measurements were carried out on five cells. Raw spectra were corrected for cosmic ray artifacts from each Raman spectrum (model: filter size: 2, dynamic factor: 10) and subsequently smoothed with a Savitzky-Golay procedure (model: order: 4, derivative: 0). Both acquisition and spectral processing were conducted using WITec Project SIX Plus software, which also provided tools for image analysis via Cluster Analysis (CA) with the centroid model and the k-means nonhierarchical analysis algorithm, in which each cluster is represented by a single mean vector. We developed the mean spectra of the cell as a whole, as well as the mean spectra of individual clusters representing a given cellular substructure, using Origin 2024 software. More details about the equipment, settings, and parameters can be found in previous works.^21–23^

### Cluster Analysis Method

Briefly, in essence, Cluster Analysis is a type of exploratory data analysis that categorizes observations into distinct groups sharing common characteristics, particularly vibrational features in our context. This method forms groups (or classes or clusters) by emphasizing similarity within each group, aiming for observations in the same group to be as alike as possible, while those in different groups should exhibit dissimilarity. The partition of n observations (*x*) into *k* (*k* ≤ *n*) clusters *S* should be done to minimize the variance (Var) according to the formula [Disp-formula eq1]:

1 $$\arg\min_{S}\overset{k}{\underset{i=1}{\sum }}\underset{x\in Si}{\sum }{∥x\mu_{i}∥}^{2}=\arg\min_{S}\overset{k}{\underset{i=1}{\sum }}\left|S_{i}\right|\mathrm{Var}S_{i}$$

where μ_*i*_ is the mean of points *S*_*i*_.

In our study, Cluster Analysis was applied to Raman images of individual single cells, ensuring that each cell was analyzed independently before comparisons across treatments were made.

The normalization was performed using Origin software (model: divided by the norm).

### Image Analysis

Advanced cell segmentation and quantitative analysis were performed using the open-source software ImageJ.^24,25^ This Java-based image processing platform, developed at the National Institutes of Health in collaboration with the Laboratory for Optical and Computational Instrumentation (LOCI, University of Wisconsin), supports visualization, editing, processing, and analysis of 8-bit color or grayscale, 16-bit integer, and 32-bit floating-point images. In the present study, ImageJ was employed to determine area measurements of interest for objects identified by intensity-based thresholding.

### Viability Tests

Tetrazolium salts have long served as reliable reagents in histochemical and cellular assays.^26,27^ Among them, the second-generation compound XTT is widely applied for the evaluation of cell viability, proliferation, metabolic activity, mitochondrial respiratory chain function, and apoptosis.^27–29^

The principle of the XTT assay is based on the colorimetric detection of cellular metabolic activity, which reflects the physiological state of cells under specific environmental conditions. During the assay, absorbance is measured at 450 nm to detect the signal generated by metabolically active cells, while 650 nm serves as a reference wavelength to correct for background. In this way, the assay provides a quantitative estimation of the metabolic activity of living cells.

Inside viable cells, tetrazolium salts are reduced by mitochondrial and cytosolic enzymes to yield colored formazan products. Since this reaction occurs only in cells with intact metabolism, the amount of formazan formed is directly proportional to the number of viable cells. A practical advantage of the XTT assay is that the reaction product is water-soluble, allowing the first reliable absorbance readings to be obtained after approximately 3 h of incubation. The sensitivity of the assay can be further enhanced by the addition of an electron-coupling reagent, PMS (*N*-methyldibenzopyrazine methyl sulfate), which accelerates the reduction of XTT and promotes formazan formation.

For each cell type, XTT tests were performed 3 and 24 h after the addition of citalopram to the cells immersed in the culture medium. Preparation for the test included proper filling of the 96-well plate according to the procedure developed at the Institute of Applied Radiation Chemistry in Lodz. Different concentrations of citalopram were selected for the test: 1, 2, 5, 10, 15, 20, 40 mg. After completing each of the 96-well plates, the samples were incubated at 37 °C. After the time from the addition of Citalopram (3, 24 h), the XTT test was performed using the Varioscan LUX Multimode Plate Reader from Thermo Fisher Scientific. A protocol was optimized specifically for the presented study. After loading the microplates into the reader, maintained at ambient temperature (20–22 °C), the samples were gently mixed before measurement. Absorbance was then recorded at 450 nm, followed by a reference reading at 650 nm. Upon completion of the measurement cycle, the plates were removed from the instrument. For all experimental variants, measurements were conducted in eight replicates.

## Results and Discussion

Given the critical role of metabolic alterations in colorectal carcinogenesis^30,31^ understanding the influence of citalopram on metabolic pathways in colon cells is of paramount importance. Cancer cells exhibit a reprogrammed metabolism characterized by increased glycolysis, enhanced glutaminolysis, and disrupted mitochondrial function to support rapid proliferation and survival.^32^ Preliminary studies suggest that citalopram may interfere with these metabolic pathways by modulating key enzymes, altering mitochondrial dynamics, and influencing oxidative stress responses. Moreover, serotonin itself plays a regulatory role in metabolic homeostasis, raising the possibility that citalopram-mediated changes in serotonin signaling may contribute to metabolic reprogramming in CRC cells.^33^

Citalopram has been shown to influence metabolic pathways in colon cells by altering mitochondrial bioenergetics and oxidative phosphorylation.^34–37^ By modulating key metabolic enzymes, such as hexokinase and pyruvate kinase, citalopram may shift cellular metabolism away from aerobic glycolysis (the Warburg effect) toward oxidative metabolism, potentially reducing the proliferative capacity of colorectal cancer cells. Additionally, citalopram’s role in regulating reactive oxygen species (ROS) production suggests that it may enhance oxidative stress in cancer cells, leading to apoptosis and reduced tumor growth.^8,38^

Figure 2 shows the schematic representation of the mechanism, proposed by Yu et al., in which serotonin modulates signaling pathways in colon cancer and SSRI antidepressant citalopram activity, reversing the Warburg effect to inhibit carcinoma by directly targeting glucose transporter protein 1 (GLUT1), respectively.^39^

**Figure 2:**
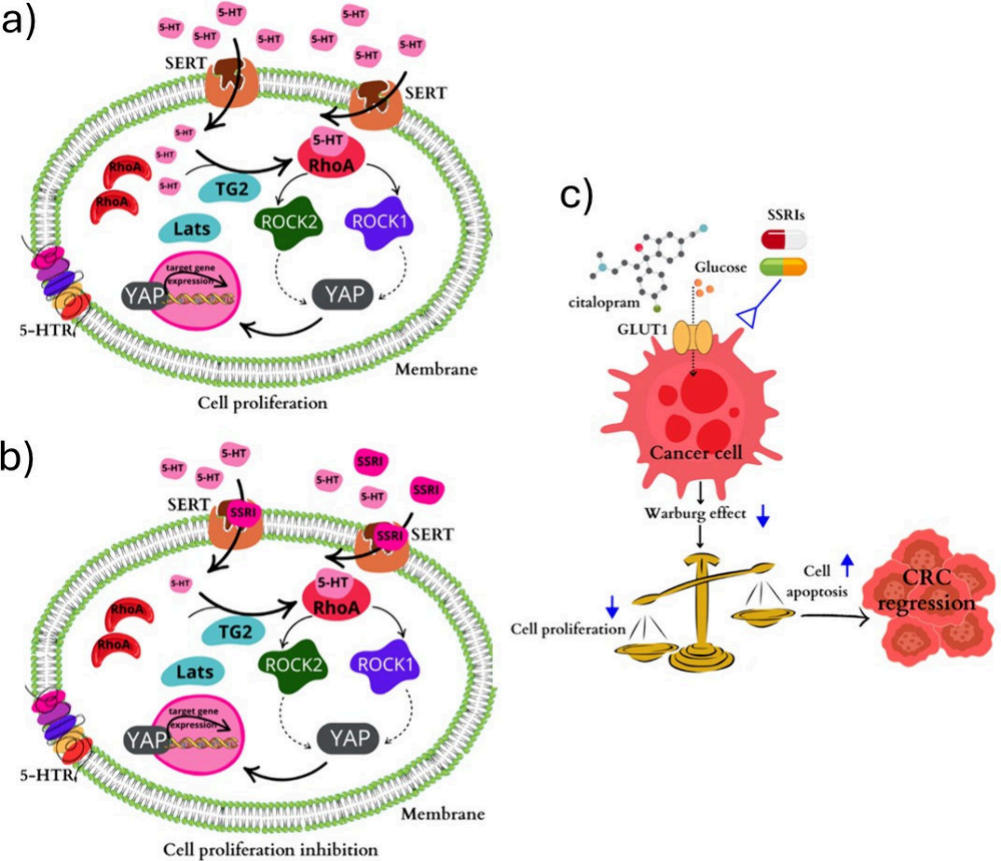
Schematic representation of the proposed mechanism by which serotonin modulates signaling pathways in colon cancer. (a) In colon cancer cells, extracellular serotonin is transported into cells by Serotonin Transporter (SERT) located in the cytomembrane and then transamidases and activates Ras Homologue Family Member A (RhoA) via serotonylation, regulated by Transglutaminase-2 (TG2). Subsequently, Rho-Associated Protein Kinase ROCK1/2 is activated, leading to the expression of Yes-Associated Protein (YAP) and its target proteins. (b) When SERT function is blocked by specific antagonists (such as citalopram), serotonin intracellular transportation is weakened to some degree, blocking the cellular signaling transduction and colon cancer cell proliferation induced by serotonin. This strategy suggests that targeting serotonin signaling may be feasible for treating colon cancer. (c) Mechanism of SSRI antidepressant citalopram activity reversing the Warburg effect to inhibit carcinoma by directly targeting GLUT1. Based on ref 39

To determine the experimental concentrations of citalopram, we completed our spectroscopic studies with XTT assays (Figures 3, 4, and 5). As illustrated in Figure 3, the addition of citalopram primarily stimulated the growth of normal human colon cells (CCD-18Co). In contrast, its addition to human colon cancer cells decreased their viability, with a more pronounced effect observed after 24 h of supplementation.

**Figure 3:**
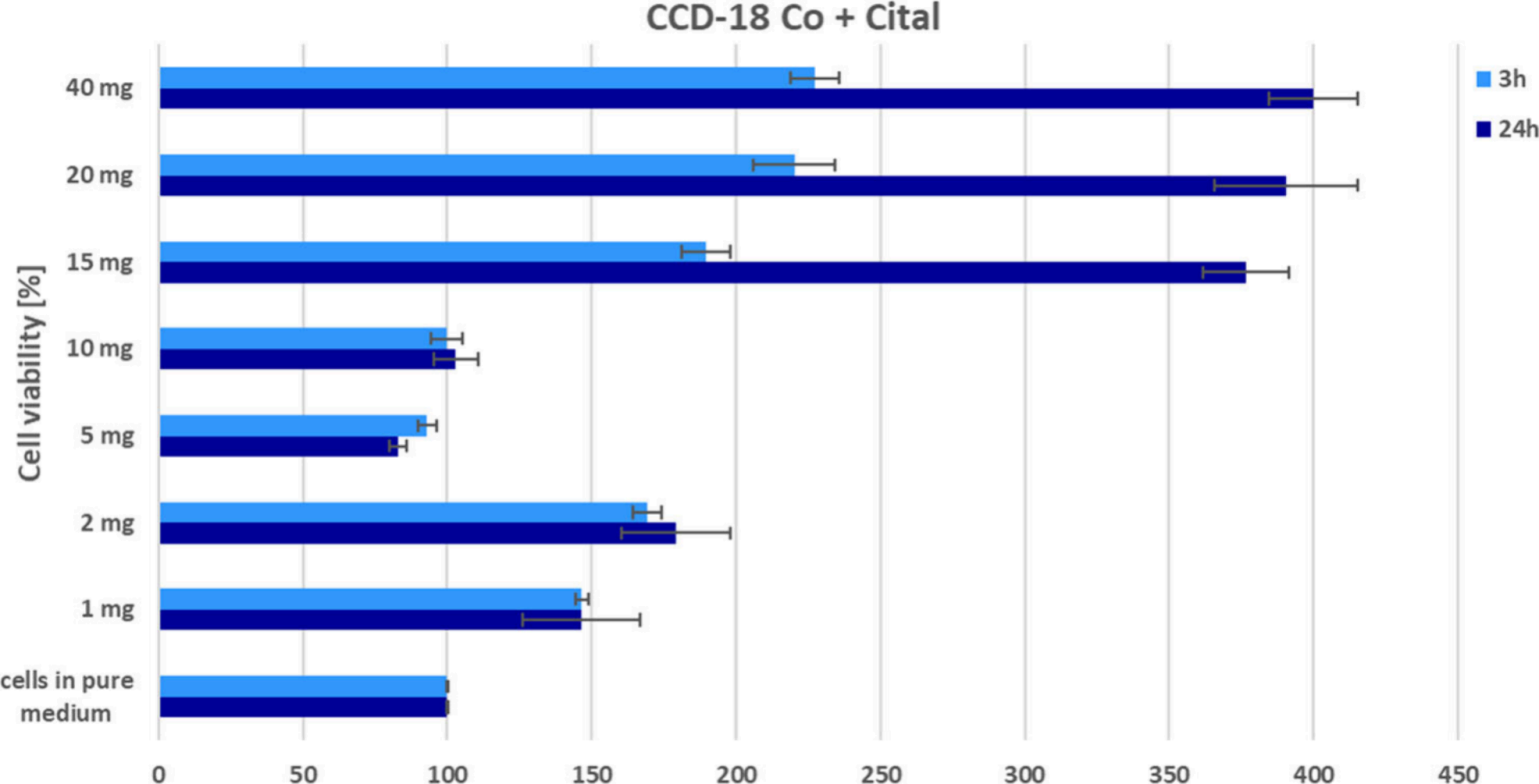
XTT data for human normal colon cells (CCD-18 Co) not supplemented and cells after adding different concentrations of citalopram for 3 and 24 h of incubation. Data are presented as mean ± SD. For the XTT viability assay, eight replicates were employed for each cell type.

**Figure 4:**
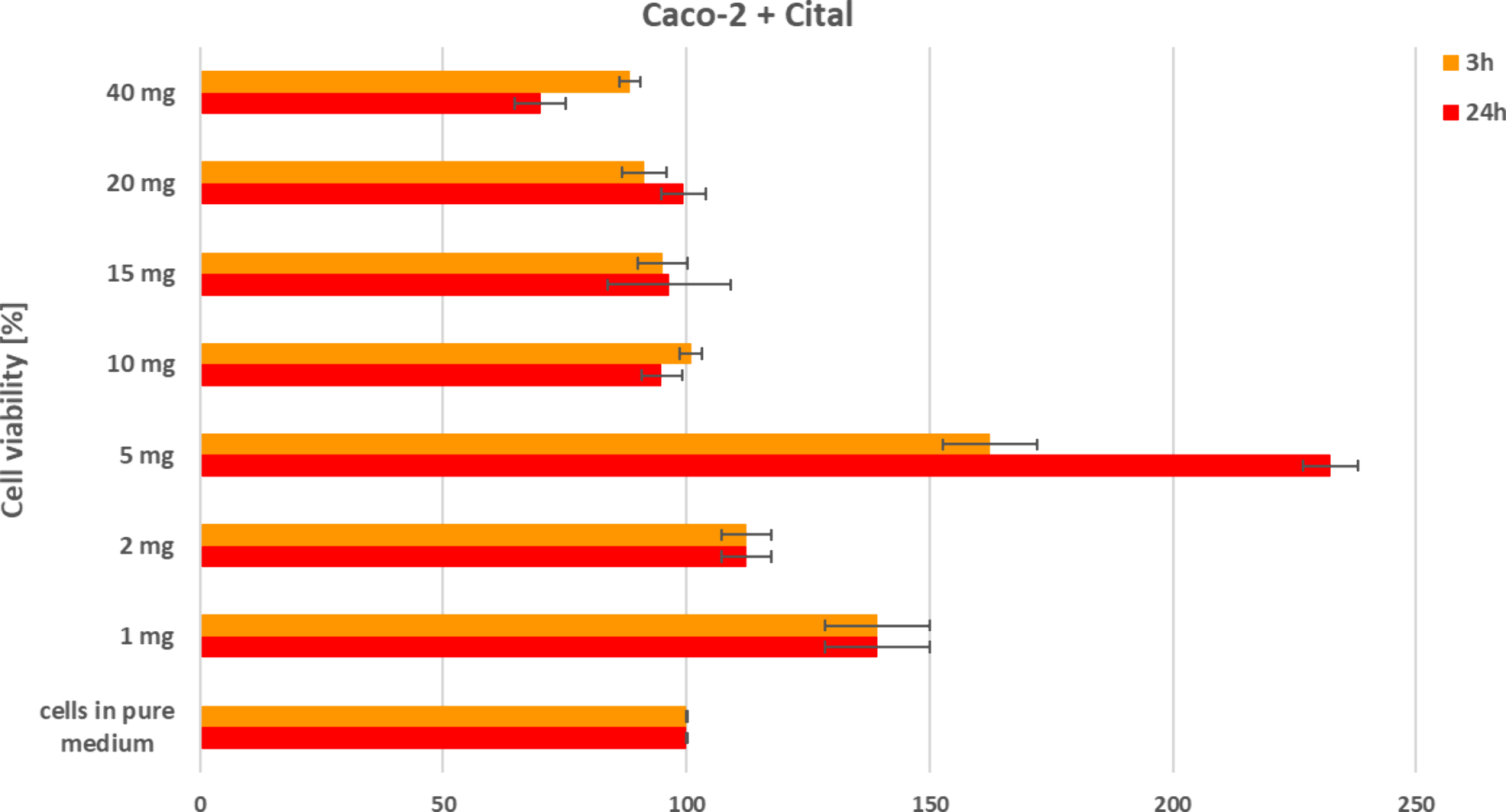
XTT data for human colon cancer G1 cells (Caco-2) not supplemented and cells after adding different concentrations of citalopram for 3 and 24 h of incubation. Data are presented as mean ± SD. For the XTT viability assay, eight replicates were employed for each cell type.

**Figure 5:**
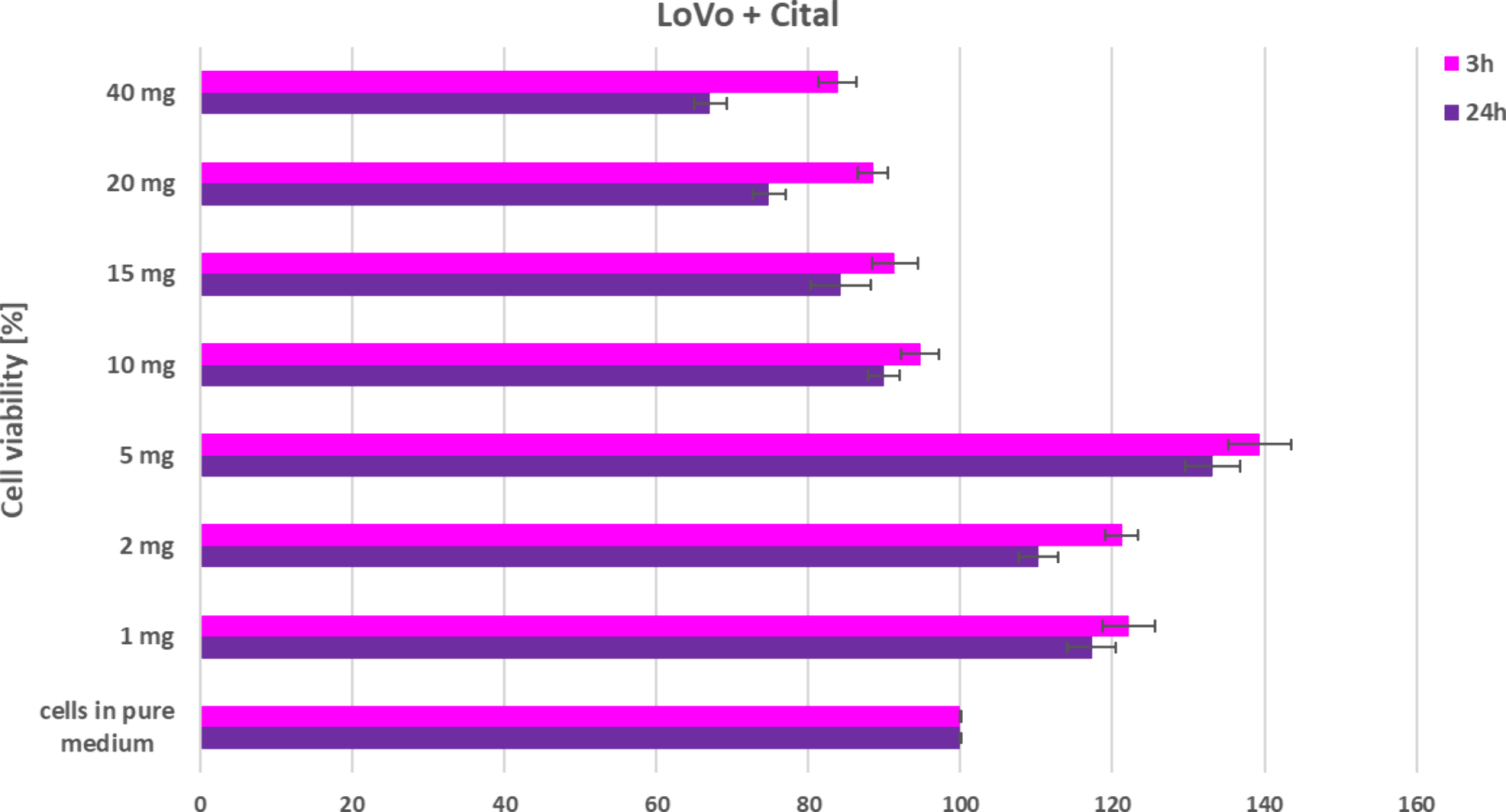
XTT data for human colon cancer G4 cells (LoVo) not supplemented and cells after adding different concentrations of citalopram for 3 and 24 h of incubation. Data are presented as mean ± SD. For the XTT viability assay, eight replicates were employed for each cell type.

Citalopram has been shown to influence the behavior of normal human colon cells. Studies indicate that its addition can stimulate the growth of normal colon fibroblasts, such as CCD-18Co cells.^40–42^ For normal colon cells, higher doses of citalopram resulted in greater cell viability. This effect was more pronounced after 24 h of supplementation, where viability reached the highest levels, particularly at 15–20 mg, while at very high concentrations (40 mg) the response appeared to plateau. In contrast, after 3 h of supplementation, the increase in viability was modest, indicating that the stimulatory effect of citalopram requires time to develop.

The delayed but marked increase after 24 h is consistent with the idea that fibroblasts absorb and respond to citalopram gradually, through the activation of intracellular signaling pathways and metabolic adaptation. In this context, the term should be understood as the transient increase in cell viability observed shortly after supplementation, followed by a decline as citalopram is gradually released and metabolized over time. This response is likely mediated through serotonin signaling pathways, which are known to play a role in cellular communication and proliferation.^43–45^ The observed stimulation suggests that citalopram may enhance certain physiological processes in normal cells, potentially contributing to tissue repair or maintenance. However, the exact mechanisms through which citalopram affects these cells, including whether this stimulation is dose-dependent or involves specific serotonin receptor subtypes, remain to be fully understood.

The XTT viability assay indicated that citalopram treatment affected the metabolic activity of colon epithelial cells, which may suggest a modulatory role of serotonin signaling in epithelial cell viability. Further studies are required to elucidate its potential impact on mucosal integrity. By promoting fibroblast growth and function, citalopram may support extracellular matrix production and enhance tissue repair mechanisms, especially under conditions of mild damage or stress. Understanding the precise mechanisms by which citalopram influences normal colon cells could have significant clinical implications. It may assist in developing therapies for gastrointestinal disorders involving impaired mucosal repair or inflammation, such as inflammatory bowel disease (IBD). Additionally, investigating the dose–response relationship and the role of specific serotonin receptors could provide valuable insights into optimizing therapeutic strategies.

The XTT assay showed that citalopram reduced the viability of colon cancer cells more strongly than that of normal colon epithelial cells. This difference may reflect the disruption of pathways supporting cancer cell proliferation. Certain serotonin receptor subtypes are overexpressed in cancer cells and promote their growth. By decreasing serotonin availability, citalopram may weaken these signaling pathways. SSRIs can disrupt indirectly the regulation of cell cycle proteins, such as cyclins and cyclin-dependent kinases, leading to a halt in proliferation.^46–49^ Investigations of viability under citalopram’s influence show that this drug can enhance the production of reactive oxygen species (ROS) in cancer cells, leading to oxidative stress.^34,36,50–54^ Due to their high metabolic activity, cancer cells are particularly vulnerable to disruptions in redox balance, which can result in cellular damage and death. The XTT assay showed that citalopram reduced the viability of colon cancer cells. This decrease in viability may indirectly suggest that citalopram could affect cancer cell interactions with the microenvironment, potentially by modulating the production of pro-inflammatory cytokines or angiogenic factors. Our research has shown that the effects of citalopram are dose- and time-dependent. The strongest effects are observed at higher concentrations and after 24 h of incubation, suggesting a cumulative impact of the drug on cancer cells.

The viability assay demonstrated that citalopram reduces the survival of LoVo colorectal cancer cells in a dose- and time-dependent manner. Increasing concentrations of the drug gradually decreased cell viability, with the most pronounced effects observed after 24 h of incubation, indicating cumulative disruption of essential cellular processes. Compared with less aggressive CRC cell line, LoVo cells exhibited a stronger reduction in viability, which may be attributed to their advanced malignant phenotype. Highly proliferative tumor cells are characterized by elevated metabolic activity and increased basal levels of ROS, making them more susceptible to disturbances in redox homeostasis.^55,56^ Citalopram has been reported to enhance ROS production, leading to oxidative damage, mitochondrial dysfunction, and subsequent impairment of ATP synthesis.^57,58^ Moreover, inhibition of serotonin-dependent signaling pathways may interfere with cell-cycle regulation and pro-survival cascades, further contributing to growth inhibition.^33,59^ These findings suggest that aggressive colorectal cancer cells are particularly vulnerable to pharmacological interference targeting metabolic balance and proliferative signaling, supporting the potential relevance of citalopram as an adjunct in anticancer strategies. Additionally, citalopram-induced ROS accumulation can damage mitochondrial membranes, impair oxidative phosphorylation, and trigger apoptosis through cytochrome-c release.^60,61^ These combined effects disproportionately impact rapidly dividing cancer cells, suggesting that interference with serotonin-dependent metabolic and signaling pathways may represent a promising approach for targeting aggressive colorectal tumors.

Overall, these data indicate that while low concentrations of citalopram may transiently stimulate LoVo cell survival by engaging pro-growth metabolic signaling, higher concentrations overcome these adaptive mechanisms and induce cytotoxicity through oxidative stress, metabolic disruption, and inhibition of serotonin-driven proliferative pathways. This supports the potential of targeting serotonin signaling in aggressive colorectal cancer phenotypes.

Moreover, the viability investigation revealed that a standard therapeutic dose of 20 mg citalopram exhibited a moderate anticancer effect, indicating that even at this level, citalopram can inhibit cancer cell proliferation. In certain clinical situations, the dose of citalopram is increased to 40 mg. XTT data confirmed that this higher dose demonstrated a stronger antiproliferative effect, likely due to more intensive modulation of cellular signaling pathways, including those influenced by serotonin. Continuous, 24-h supplementation with citalopram consistently results in lower cancer cell viability. This suggests that both the dosage intensity and the duration of exposure are critical factors in maximizing the drug’s anticancer potential. By ‘dosage intensity’ we refer to the amount of drug administered relative to a given unit of time, which reflects not only the absolute dose but also the frequency of administration.

In summary, our findings indicate that citalopram exposure may be linked to metabolic alterations in both normal and cancerous colon cells, although the exact mechanisms remain to be established. In normal cells, the observed spectral changes suggest only moderate metabolic effects, which could involve drug metabolism or interactions influencing its bioavailability. Possible consequences might include adjustments in energy balance or nutrient utilization, reflecting an adaptive response to the treatment. In contrast, cancerous cells appear to undergo more pronounced reprogramming, and one potential explanation could be their altered expression of drug-metabolizing enzymes such as cytochrome P450s. Additionally, the distinct redox environment of malignant cells, characterized by elevated ROS, may contribute to differences in citalopram metabolism and impact its efficacy or toxicity. These interpretations should be regarded as working hypotheses that provide directions for further biochemical validation and mechanistic studies, rather than definitive conclusions.

Figure 6 shows the examination of colon cells through Raman spectroscopy and imaging, focusing on the regions associated with the endoplasmic reticulum (ER), and lipid droplets (LD).

**Figure 6:**
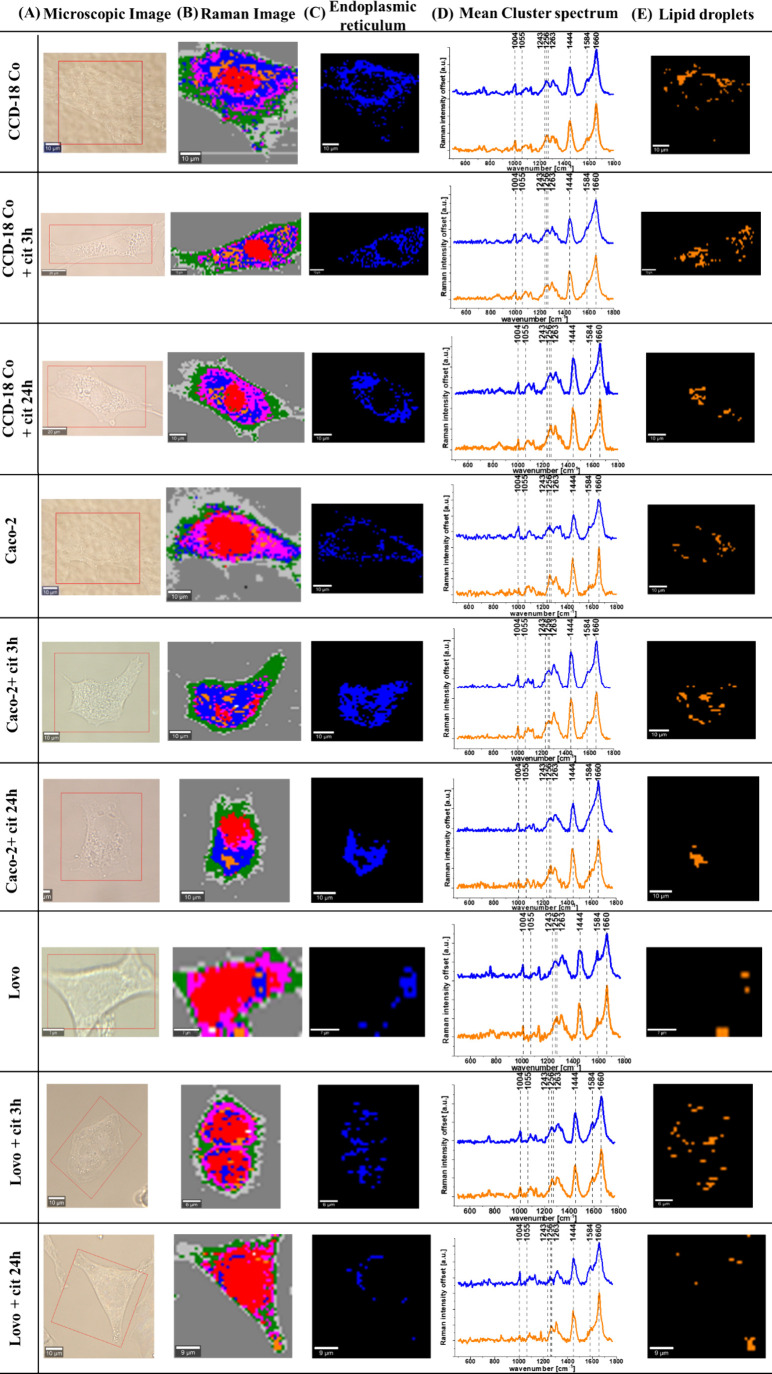
Microscopic image of a single cell (A), Raman images constructed based on Cluster Analysis (CA) (B), the single clusters wherein blue corresponds to ER (C) and orange corresponds to LDs (E) identified by using CA, and the mean Raman spectra typical for selected clusters identified by using CA (D). The figure presents results for human normal colon cells CCD-18 Co and human colon cancer Caco-2 and LoVo cells without any supplementation and upon citalopram supplementation for 3 and 24 h for 20 mg. All cells were measured in PBS. The scale bar represents 10 μm. The colors of the spectra correspond to the colors of the clusters. For each cell type and experimental condition, Raman spectroscopy measurements were obtained from five individual cells.

We decided to focus on the ER and LDs because ER is a major hub for fatty acids (FAs) metabolism by being implicated in uptake of exogenous FAs, FAs *de novo* synthesis, lipids elongation, and desaturation.^62–68^ It is important to note that in all cells, ER maintains contact with the membranes of various organelles, including mitochondria, the nucleus, LDs, and peroxisomes.^69–75^ These interactions facilitate the efficient transfer of FAs substrates and enzymes between compartments.^69–75^ Simultaneously, LDs serve several critical functions in human cells: a) energy storage: LDs store neutral lipids, primarily triacylglycerols (TAGs) and cholesteryl esters, which act as energy reserves.^76–79^ When energy is needed, these lipids are hydrolyzed into FAs, which are then utilized in mitochondrial β-oxidation to generate ATP; b) lipid homeostasis and detoxification: LDs help regulate lipid balance within the cell and can sequester excess or potentially toxic lipids, thereby preventing lipotoxicity. They also contribute to protection against oxidative stress by storing oxidized lipids; c) membrane lipid supply: LDs function as a lipid reservoir, providing essential components for the synthesis and repair of cellular membranes, particularly during periods of rapid cell growth or membrane remodeling; d) protein sequestration and degradation: LDs can associate with various proteins, including misfolded or damaged ones, and may facilitate their storage or degradation, thereby supporting cellular proteostasis;^80^ e) host–pathogen interactions: certain pathogens, such as viruses and bacteria, exploit LDs to support their replication or to evade host immune responses;^81–83^ f) signaling and metabolism: LDs participate in lipid signaling pathways and metabolic regulation.^83–85^ They interact dynamically with other organelles, including the ER and mitochondria, to coordinate cellular metabolic processes.

Organelles were identified using Cluster Analysis, which enables unbiased segmentation of Raman maps and facilitates precise localization of metabolic alterations within distinct subcellular compartments. Clusters in the Raman maps were assigned to specific cellular components based on their characteristic spectral profiles. The following Raman bands were used for this purpose: 1004 cm^–1^ (phenylalanine ring breathing, indicative of protein content), 1055 cm^–1^ (C–C stretching of lipids), 1243, 1256, 1263 cm^–1^ (Amide III bands, associated with protein secondary structure), 1444 cm^–1^ (CH_2_/CH_3_ bending, common in lipids), 1584 cm^–1^ (C = C stretching in aromatic amino acids and nucleic acids), 1660 cm^–1^ (Amide I, reflecting protein backbone structure), 2854, 2930 cm^–1^ (CH_2_/CH_3_ stretching vibrations, characteristic of lipid-rich regions). Based on these marker bands, the endoplasmic reticulum (ER) clusters were primarily identified by the Amide III and I bands (1243–1263 cm^–1^ and 1660 cm^–1^) and the 1004 cm^–1^ peak, reflecting the protein-rich nature of the ER, as well as by characteristic lipid-associated bands, consistent with the mixed protein–lipid composition of this organelle. Lipid droplet (LD) clusters were characterized by the strong lipid-associated bands at 1055, 1444, 2854, and 2930 cm^–1^. Additional bands such as 1584 cm^–1^ provided complementary information for distinguishing nucleic acid- and protein-rich regions within cells. The spectral assignment was guided by previously published studies on Raman spectroscopy of biological samples.^86–104^

CA was performed on individual single-cell Raman maps, ensuring that assignments were consistent across cells of the same type and treatment. The characteristic spectral differences between ER and LDs clusters, including peak positions and relative intensities, were used to accurately distinguish these regions within the cells.

To determine the most important frequencies that differentiate normal and cancerous human colon cells, as well as cancer cells of varying aggressiveness, in the absence and presence of citalopram, pairwise PCA (Principal Component Analysis) was performed. Figure 7 shows the pairwise PCA results based on mean Raman spectra from CCD-18 Co, Caco-2, and LoVo cell lines, either untreated or treated with citalopram for 3 and 24 h for ER, including PCA score plots and biplot representations. The corresponding three-dimensional PCA score plots are provided in the Supporting Information (Figure S1).

**Figure 7:**
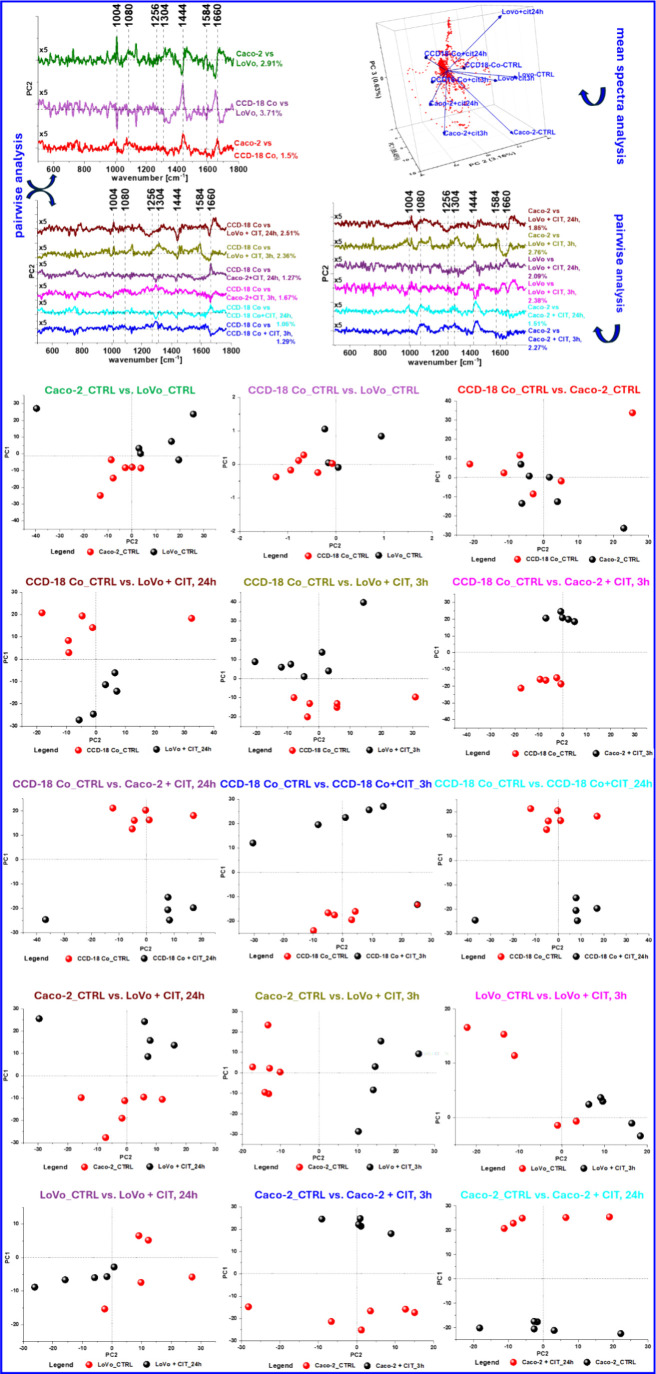
Pairwise PCA of mean Raman spectra acquired in the fingerprint region from normal CCD-18 Co and cancerous Caco-2 and LoVo human colon cells, either untreated or supplemented with citalopram for 3 and 24 h. Mean spectra, PCA score plots, and corresponding PCA biplots illustrating spectral differences between cell types and treatment conditions. Raman measurements were performed using a 532 nm laser. For each experimental condition, spectra from five individual cells were collected and averaged before PCA. The fingerprint region covered the 600–1800 cm^–1^ spectral range. Panel labels correspond to the comparisons indicated below each plot.

Figure 7 and S1 demonstrate that the PCA algorithm effectively visualizes biochemical changes induced by drug exposure, as well as those occurring in nonsupplemented cells.

PCA reveals that the separation among control cell populations is not fully resolved, indicating partial overlap in their global molecular profiles. In contrast, the discrimination among the three investigated cell types improves progressively with increasing tumor aggressiveness. The most pronounced separation is observed between the nonsupplemented G1 and G4 cancer cells, reflecting substantial intrinsic differences in their biochemical composition and cellular physiology. These differences are consistent with malignancy-associated alterations in metabolic activity, membrane organization, and intracellular regulation, which become more prominent as tumor aggressiveness increases and are effectively captured by the principal components.

Cell supplementation leads to a further enhancement of class separation, with the degree of discrimination increasing with supplementation time and reaching its maximum after 24 h of citalopram exposure. This observation is consistent with the fact that normal colon cells (CCD-18 Co, low-aggressiveness (G1) cancer cells (Caco-2), and highly aggressive (G4) cancer cells (LoVo) rely on distinct proliferation mechanisms and metabolic strategies. As a result, their responses to external perturbations differ, and prolonged supplementation allows these response-specific molecular changes to accumulate, thereby improving their detectability in multivariate space.

From a biological standpoint, the enhanced separation following citalopram supplementation can be attributed to fundamental differences in cellular architecture and physiology between normal and cancerous cells. Malignant transformation is accompanied by metabolic reprogramming, altered mitochondrial function, changes in membrane lipid composition, and dysregulation of intracellular signaling pathways. These features not only distinguish cancer cells from normal cells but also vary systematically with tumor aggressiveness, giving rise to distinct baseline molecular phenotypes for G1 and G4 cells.

Citalopram exposure further amplifies these differences by interacting with cellular membranes and intracellular organelles, including mitochondria. Due to variations in membrane permeability, organelle function, and metabolic demands, individual cell types exhibit distinct uptake, distribution, and intracellular responses to citalopram. Highly aggressive cancer cells, characterized by increased metabolic fluxes and structural plasticity, are particularly susceptible to such perturbations. Consequently, supplementation induces cell-type-specific biochemical rearrangements, such as changes in lipid organization, energy metabolism, and redox balance, which are reflected in the improved class separation observed in the PCA score plots.

As illustrated in Figure 7, PC1 predominantly drives the differentiation of multiple control and citalopram-treated cell populations, differentiating (CCD18-Co_CTRL vs LoVo+CIT, 24h), (CCD18-Co CTRL vs LoVo+CIT, 3h), (CCD18-Co CTRL vs Caco-2+CIT, 3h), (CCD18-Co CTRL vs Caco-2+CIT, 24h), (CCD18-Co_CTRL vs CCD18_Co+CIT_3h), (CCD-18-Co CTRL vs CCD18 Co+CIT 24h), (Caco-2_CTRL vs LoVo+CIT, 24h), (Caco-2_CTRL vs Caco-2+CIT, 3h), and (Caco-2 CTRL vs Caco-2+CIT, 24h). Simultaneously, PC2 contributes to the differentiation of (Caco-2 CTRL vs LoVo+CIT, 3h), (LoVo CTRL vs LoVo+CIT, 3h), and (LoVo CTRL vs LoVo+CIT, 24h). Together, these results indicate that PC1 captures the dominant variance associated with both cell type and supplementation status, while PC2 reflects additional variance related to cell-line-specific and time-dependent responses to citalopram exposure.

Importantly, the PCA loadings define which specific Raman bands contribute most strongly to the variance captured by the principal components. Moreover, the figure confirms the mutual relationships between the corresponding PCA loadings and visually confirms the spectral differentiation between the experimental groups. On this basis, we selected the most prominent bands for subsequent Raman band ratio analysis to obtain biologically meaningful insights.

Characteristic Raman bands observed at 1004, 1054–1080, 1243–1256, 1292, 1444, 1584, and 1660–1684 cm^–1^ correspond to molecular vibrations associated with aromatic amino acids (phenylalanine), phosphate groups (RNA/DNA, phospholipids), protein structures (Amide III and I), and membrane lipids (CH_2_ deformation).^86^ In CCD-18Co cells treated with citalopram, significant changes in intensity were observed at 1256, 1444, and 1660 cm^–1^ at both 3 and 24 h. All these observations suggest alterations in protein content and membrane lipid dynamics, potentially due to the lipophilic nature of citalopram interacting with cell membranes or modulating biosynthetic pathways through intracellular signaling or transcriptional regulation.

More pronounced differences were observed in Caco-2 cells treated with citalopram. After only 3 h, additional peaks appeared at 1243, 1292, and 1584 cm^–1^, indicating changes in protein secondary structure and possibly the activation of transcriptional processes.^86^ The heightened response of the cancer cell line may reflect its altered metabolism, increased demand for regulatory proteins, and greater sensitivity to environmental stressors such as pharmacological agents.

These results indicate that citalopram exerts significant biochemical effects on non-neuronal cells, extending beyond its canonical role as an SSRI. These effects include direct alterations in membrane architecture, protein composition, and gene expression.

In LoVo cells, citalopram treatment also induced measurable spectral alterations, although the pattern differed from both CCD-18Co and Caco-2 cells. After 3 h of supplementation, distinct intensity changes were observed within the 1054–1080 cm^–1^ and 1444 cm^–1^ regions, suggesting early modifications in phospholipid organization and CH_2_ bending modes associated with membrane remodeling.^86^ These shifts are consistent with the highly dynamic membrane turnover characteristic of metastatic colorectal cancer cells. At 24 h, additional variations became evident at 1584 and 1660–1684 cm^–1^, which correspond to alterations in aromatic amino acid content and Amide I vibrations, respectively.^86^ Such changes indicate remodeling of protein secondary structure, potentially driven by stress-responsive signaling pathways or increased proteostasis demands. The temporal progression of spectral modifications suggests that LoVo cells exhibit a delayed but progressively intensifying biochemical response, likely consistent with metabolic dysregulation potentially linked to oxidative stress, as suggested by alterations in lipid-associated Raman bands, and heightened susceptibility to perturbations in membrane integrity and protein biosynthesis.^105,106^ Taken together, the responsiveness of LoVo cells to citalopram may be associated with their metastatic phenotype, elevated proliferation rate, and a greater reliance on lipid-mediated signaling cascades.

The PCA biplot score plot provides a multidimensional representation of sample separation in the reduced feature space together with the loading vectors of characteristic Raman bands. Normal CCD-18Co, Caco-2, and LoVo cells occupy distinct score regions, indicating pronounced biochemical divergence driven by differences in cellular components. This spatial segregation reflects the intrinsic metabolic phenotypes of normal versus malignant cells.^107,108^ The displacement of citalopram-treated samples relative to untreated controls illustrates treatment-dependent metabolic reprogramming.

Raman spectroscopy has proven to be a sensitive method for detecting these subtle but meaningful metabolic and physiological changes in both normal and cancerous cells.^87–93,96–98,102–104,109–122^ The data highlight the utility of this technique in exploring the off-target effects of psychotropic drugs and support its application in cellular pharmacodynamics and metabolic profiling.^87–93,96–98,102–104,109–122^

Figure 8 presents box charts with normal distribution lines for selected Raman band intensity ratios, which were selected based on PCA analysis results.

**Figure 8:**
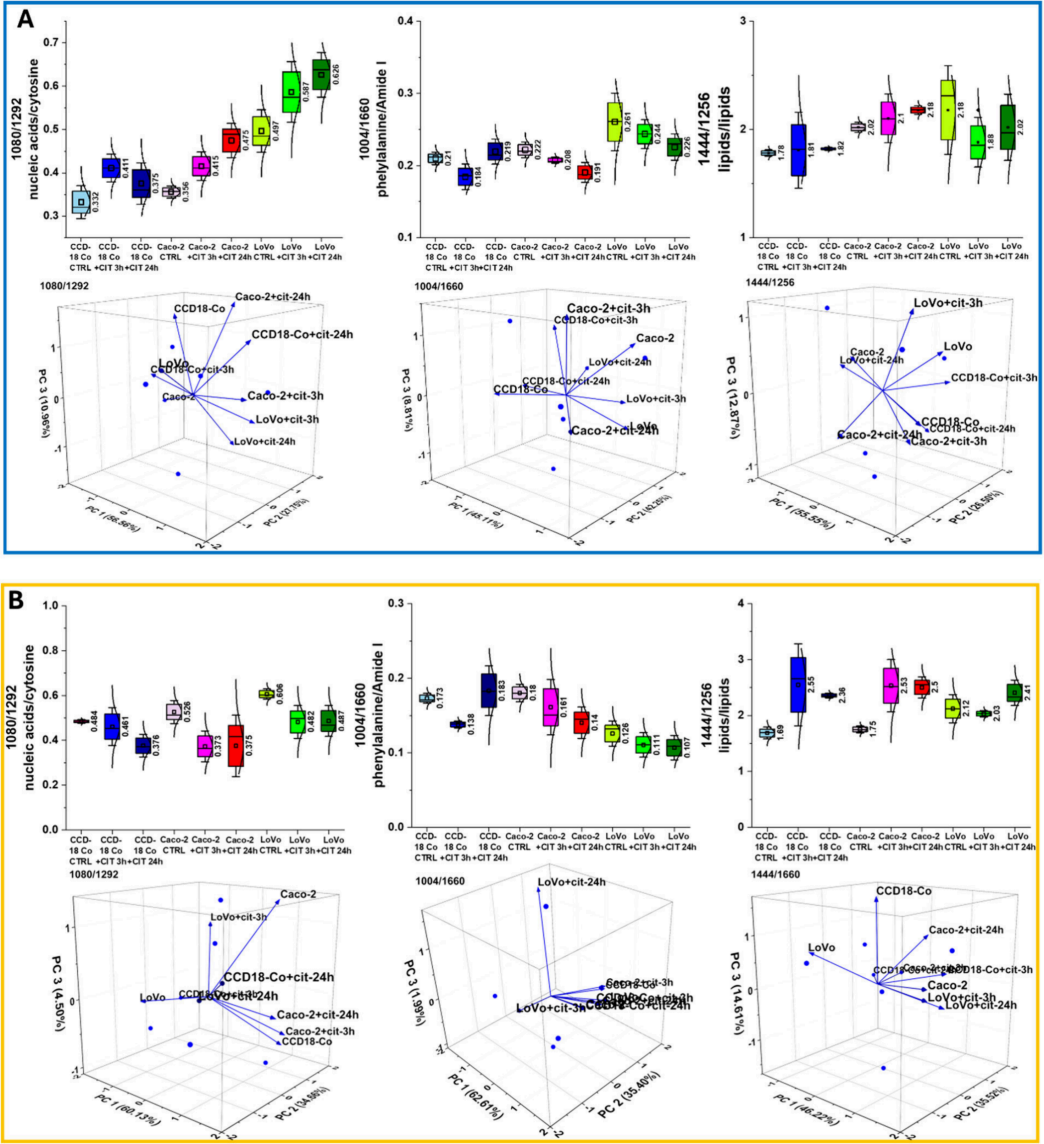
Box charts for the Raman band intensity ratio for selected Raman bands: 1080/1292 corresponding to nucleic acids/cytosine, 1004/1660 corresponding to proteins/proteins, and 1444/1256 corresponding to lipids/lipids for ER (A, blue cluster in RI data), and LDs (B, orange cluster in RI data). Data are presented as mean ± SD (horizontal line inside the box), and the data distribution is presented as corresponding PCA score biplots. For each cell type and experimental condition, Raman spectroscopy measurements were obtained from five individual cells.

The figure illustrates the analysis of biochemical composition in Caco-2, LoVo, and CCD-18Co cells, focusing on the ER and LDs. The ratios of selected spectral bands were examined to assess changes in nucleic acids, proteins, and lipids under control conditions and after exposure to citalopram for 3 and 24 h.

To further elucidate the biochemical alterations induced by citalopram at the subcellular level, ratiometric Raman band intensity analysis was performed for key molecular groups in ER and LDs.

As we have mentioned above, ER and LDs are critical organelles involved in the metabolic regulation of both cancerous and normal colon cells. Their function and biochemical composition reflect the distinct metabolic demands and behaviors of these two cell types. In LoVo and Caco-2 cells, which are characterized by rapid proliferation and high metabolic activity, the ER and LDs are highly engaged in protein synthesis, lipid biosynthesis, and managing cellular stress responses such as the unfolded protein response (UPR).^123–130^ These processes are upregulated to meet the demands of tumor growth. In contrast, in CCD-18Co cells, they operate under tighter control, supporting steady-state cellular functions such as normal protein folding and membrane maintenance.^131–135^

To probe the molecular features of these organelles, specific Raman bands, based on PCA, were selected, taking into account their well-established associations with key cellular biomolecules:

The 1080/1292 cm^–1^ ratio reflects the relative abundance of phosphate backbones (1080 cm^–1^) and cytosine ring vibrations (1292 cm^–1^). It is indicative of nucleic acid content and organization, particularly DNA/RNA levels. These are highly relevant in cancer cells, which have elevated nucleic acid synthesis due to increased transcription and replication.

In the ratio 1004/1660 cm^–1^, the 1004 cm^–1^ band corresponds to phenylalanine ring breathing, a relatively stable marker of total protein content, while the 1660 cm^–1^ band is associated with Amide I, mainly arising from the C = O stretch of the peptide bond and sensitive to protein secondary structure. This ratio provides insights into protein expression and folding dynamics, which are often altered in cancer due to ER stress, enhanced protein synthesis, and misfolded protein accumulation.

In the ratio 1444/1266 cm^–1^, both bands are linked to lipid vibrational modes: 1444 cm^–1^ relates to CH_2_ bending and reflects saturated lipid content, while 1266 cm^–1^ is connected to = C–H in-plane bending, more prominent in unsaturated lipids. The ratio between these bands serves as a marker of lipid composition and saturation, which is especially significant in cancer cells with upregulated lipid biosynthesis, membrane remodeling, and lipid storage in LDs.

By analyzing these specific Raman band ratios, one can effectively trace the molecular and functional differences between cancerous and normal cells at the subcellular level. The selected spectral markers are tightly linked to core metabolic processes - nucleic acid turnover, protein homeostasis, and lipid metabolism - that are fundamentally altered during tumorigenesis and are reflected in the behavior of organelles like the ER and LDs.

### Nucleic Acids/Cytosine (1080/1292 cm^–1^)

In the ER, a significant increase in the 1080/1292 ratio was observed in Caco-2 cells after 24 h of citalopram exposure, suggesting enhanced nucleic acid-related activity, likely as a stress response leading to upregulated transcription or replication. The effect was even more pronounced in LoVo cells, which exhibit a higher degree of aggressiveness compared to Caco-2 cells. In contrast, CCD-18Co cells showed a transient increase at 3 h, followed by a marked reduction at 24 h. This biphasic response in normal cells might indicate an initial activation of stress response pathways, followed by adaptation and repression of nucleic acid metabolism to maintain homeostasis.

In LDs, the nucleic acid-related ratio decreased markedly in Caco-2 cells upon citalopram exposure at both time points, possibly reflecting redistribution of nucleic acid components or suppression of transcriptional activity within lipid-rich domains. A similar tendency was noted in LoVo cells, which are characterized by higher aggressiveness. CCD-18Co cells showed a similar downward trend, particularly after 24 h, indicating a reduced engagement of these compartments in nucleic acid metabolism under drug-induced stress.^136–143^

These observations suggest that citalopram exerts a compartmentalized and cell-type-specific effect on nucleic acid dynamics. While cancer cells respond with increased ER-driven transcriptional activity, possibly supporting survival or proliferation under stress, normal cells reduce their metabolic burden through feedback inhibition mechanisms.

### Proteins: Phenylalanine/Amide I (1004/1660 cm^–1^)

The protein-related ratio in the ER declined significantly in Caco-2 cells after 3 and 24 h, implying disrupted protein synthesis or increased proteolytic degradation. This may reflect unfolded protein accumulation and ER stress, consistent with UPR (unfolded protein response) activation. This pattern was also evident in LoVo cells, known for their more aggressive phenotype, although the values remained higher than those observed in G1-grade cells. In contrast, CCD-18Co cells displayed an initial decrease, followed by a marked increase after 24 h, indicating a recovery of protein synthetic capacity and possible resolution of ER stress.^136–143^

In LDs, a strong decrease in the protein ratio was again seen in Caco-2 cells, aligning with the suppression of active translation or protein aggregation in lipid compartments. The lowest ratio values among cancer cells were observed in the aggressive LoVo line. CCD-18Co cells showed an opposite trend, with increased protein content in LDs after 24 h, potentially indicative of adaptation via compartmentalization of stress-related proteins or enhanced biosynthesis.

These findings reflect a divergent regulation of proteostasis under citalopram exposure, with impaired protein turnover in malignant cells and preserved or even adaptive protein regulation in nontransformed fibroblasts. This divergence could underlie differences in drug sensitivity and cell viability.^136–143^

### Lipids (1444/1256 cm^–1^)

Lipid-related ratios in the ER increased significantly in Caco-2 cells after 3 h, with a plateau at 24 h. This suggests enhanced lipogenesis or membrane remodeling, potentially linked to ER membrane expansion during stress. In LoVo cells, the initial increase was followed by a slight decline. CCD-18Co cells, however, showed an initial decrease followed by a partial rebound, indicating a more controlled lipid response to preserve membrane integrity.^144–147^

In LDs, LoVo and Caco-2 cells exhibited a pronounced and sustained increase in lipid signal intensity, with the highest values after 24h, pointing to LD accumulation or altered FA metabolism. The rise in CCD-18Co cells, although delayed, supports a more gradual adaptation involving lipid storage or buffering against oxidative stress.^148–152^

These alterations highlight a strong lipidomic remodeling driven by citalopram, especially in cancer cells, potentially involving impaired β-oxidation, increased *de novo* lipogenesis, and accumulation of neutral lipids. Such shifts may contribute to membrane dysfunction or metabolic inflexibility.^148,150,153–158^

Collectively, these findings reveal distinct biochemical signatures in response to citalopram that are dependent on both the cell type and the subcellular compartment affected. LoVo and Caco-2 cells, characterized by high basal metabolic activity, respond to citalopram with increased transcription, impaired proteostasis, and substantial lipid accumulation - hallmarks of metabolic stress and potentially apoptotic signaling.^31,59,159–161^ The simultaneous rise in nucleic acids and lipids in ER may point to citalopram-induced ER stress and dysfunction of proteolipid homeostasis.

In contrast, CCD-18Co cells exhibit a more balanced and reversible response, marked by initial suppression followed by partial recovery across all biochemical axes. These dynamics reflect a higher resilience to pharmacological perturbation, likely due to intact regulatory pathways that limit cytotoxicity and promote metabolic reprogramming.

All these Raman spectroscopic insights confirm that citalopram induces profound shifts in cellular metabolism and structure, particularly affecting organelles involved in protein folding, lipid storage, and nucleic acid regulation. The implications extend to understanding off-target effects of SSRIs, the metabolic vulnerability of cancer cells, and the potential of Raman spectroscopy for assessing drug-induced cellular stress *in situ*.

Based on Raman imaging data, we also performed the analysis of morphological changes associated with citalopram supplementation. Table 1 and Figure 9 show how citalopram influences the areas of ER and LDs after 3h or 24h (data were obtained using ImageJ software).

**Figure 9:**
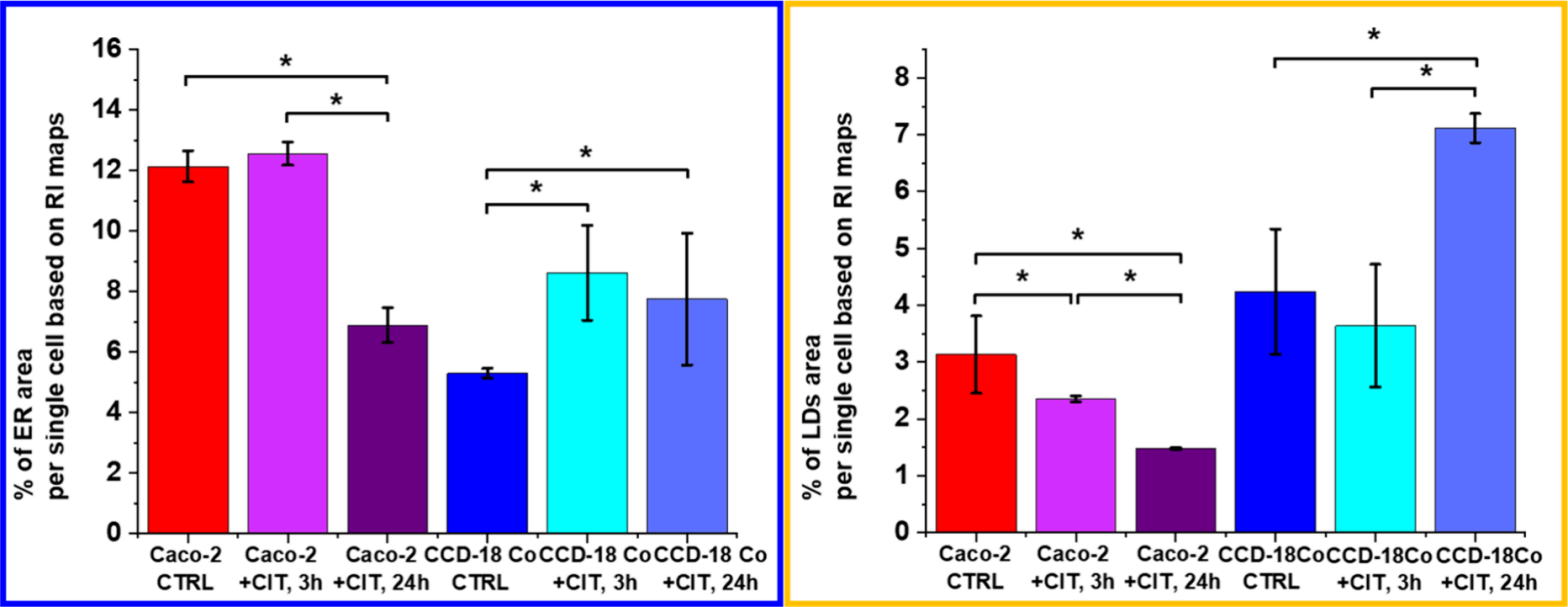
Percentage of ER (blue frame) and LD (orange frame) areas without and after adding citalopram for 3 and 24 h. Data are presented as mean ± SD. The statistically significant results, based on ANOVA analysis, have been marked with an asterisk (*p ≤ 0.05). For each cell type and experimental condition, Raman spectroscopy measurements were obtained from five individual cells.

**Table 1:**
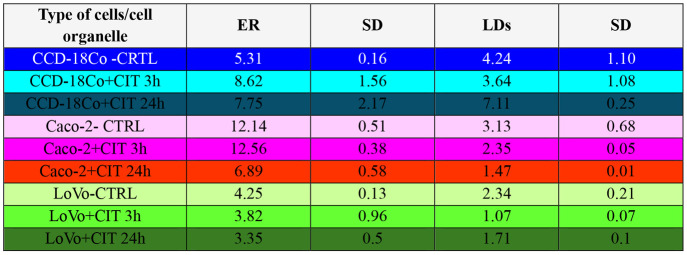
Percentage of Different Human Colon Cell Organelle Areas Per Single Cell Based on RI Maps

Table 1 presents the areas of various ER and LDs in human colon cells, quantified using Raman imaging maps.

In detail, in the case of the ER, citalopram supplementation for 24 h led to a notable decrease in its area in Caco-2 cells, dropping from approximately 13% in the control to around 7%. This suggests a strong disruptive effect of citalopram on ER integrity and function in these cancerous cells. The analysis of ER surface area in LoVo cells revealed a clear decreasing trend following supplementation, with a total reduction of 21.18%. Notably, LoVo cells exhibited the lowest ER surface area values among the tested cell lines. Despite their aggressive phenotype, the ER surface area in LoVo cells remained lower than that observed in G1-grade cells. These findings suggest that ER remodeling in LoVo cells is responsive to external stimuli and may reflect underlying mechanisms associated with cellular aggressiveness and metabolic adaptation. In contrast, the CCD-18Co cells showed an overall increase in ER area at both 3 and 24 h compared to the control, with only a slight decrease between the two time intervals. The ER plays a critical role in protein and lipid synthesis, calcium storage, and detoxification.^162,163^ Therefore, a reduction in ER area may indicate ER stress, likely triggering the unfolded protein response pathway and impairing the biosynthetic and detoxifying capacities of the cell. The more significant effect in LoVo and Caco-2 cells suggests that cancer cells are more susceptible to citalopram-induced ER disruption.

Regarding LDs, LoVo and Caco-2 cells showed a decrease in LD area following citalopram treatment. This may reflect impaired lipid metabolism or a reduction in lipid storage capacity. In contrast, CCD-18Co cells responded differently - the LD area increased significantly after citalopram treatment, especially at the 24-h mark, while a slight decrease was observed following 3 h of supplementation. LDs serve as energy reservoirs and play a role in detoxification by sequestering lipophilic toxic compounds.^80,85,164,165^ The observed increase in LDs in normal fibroblasts may therefore represent a protective response, aiming to neutralize and isolate citalopram or its metabolites.

In summary, citalopram appears to exert a stronger cytotoxic effect on cancerous LoVo and Caco-2 cells, as evidenced by reduced ER and LD areas. Meanwhile, normal CCD-18Co fibroblasts seem to activate adaptive mechanisms, particularly by increasing LDs formation. These findings underscore the differential sensitivity of normal and cancerous cells to induced stress, with potential implications for both toxicology and targeted therapies.

## Conclusions

The metabolism of citalopram in human colon cells, both normal and cancerous, is influenced by a range of factors, including enzyme expression, cellular redox state, and metabolic reprogramming. While normal cells metabolize citalopram through conventional oxidative and conjugative processes, cancer cells may exhibit altered metabolism due to the reprogramming of cellular pathways and the overexpression of specific metabolic enzymes.

This study demonstrates that citalopram, a selective serotonin reuptake inhibitor exerts distinct and compartment-specific biochemical effects on both normal (CCD-18Co) and cancerous (Caco-2 (G1) and LoVo (G4)) colon-derived cells.

Using ratiometric Raman spectroscopy, we identified significant alterations in nucleic acid, protein, and lipid profiles within the ER and LDs, revealing critical insights into drug-induced metabolic remodeling. The biplot score plot integrates molecular signatures with functional metabolic trajectories, underscoring the utility of Raman spectroscopy as a sensitive probe of pharmacologically induced biochemical dynamics in heterogeneous cellular systems.

LoVo and Caco-2 cells responded to citalopram with pronounced increases in nucleic acid-related signals in the ER, reduced protein ratios, and substantial lipid accumulation in both the ER and LDs. These changes are consistent with the induction of endoplasmic reticulum stress, altered lipid metabolism, and disrupted proteostasis - hallmarks of cellular distress and potential apoptosis initiation in metabolically active tumor cells.

Conversely, CCD-18Co cells exhibited a more adaptive and temporally regulated response. An early decrease followed by partial normalization for nucleic acid and protein ratios suggests a controlled stress response, while delayed but moderate lipid accumulation indicates a protective mechanism through lipid storage or membrane remodeling. This resilience underscores the differential susceptibility of nonmalignant cells to SSRI-induced perturbations.

Our results indicate that exposure to citalopram can influence the organization of cellular structures in both malignant and nonmalignant colon cells, although the nature of these changes appears to differ. In LoVo and Caco-2 cells, we observed alterations consistent with a reduction of ER regions and a lower abundance of LDs, which may reflect a disturbance in metabolic balance. In contrast, CCD-18Co fibroblasts displayed an opposite trend, with an expansion of LD areas that could point toward a compensatory or protective adjustment. Taken together, these outcomes suggest that cancer cells and normal fibroblasts respond to citalopram in distinct ways, though further studies are needed to elucidate the underlying mechanisms fully.

Altogether, our findings highlight the capacity of citalopram to modulate key metabolic pathways in a cell-type-specific manner. They underscore the utility of Raman spectroscopy as a powerful, label-free tool for monitoring subcellular drug effects *in situ* at high spatial resolution. These insights may contribute to a deeper understanding of SSRI off-target actions and pave the way for their repurposing or optimization in cancer therapeutics and precision medicine.

## Supplementary Material



## Notes

Pairwise three-dimensional PCA score plots (PC1–PC3) in the fingerprint region from all investigated cell lines (PDF)

## References

[1] Legge D. N., Collard T. J., Stanko E., Hoskin A. J., Holt A. K., Bull C. J., Kollareddy M., Bellamy J., Groves S., Ma E. H., Hazelwood E., Qualtrough D., Amulic B., Malik K., Williams A. C., Jones N., Vincent E. E. (2024). Identifying Targetable Metabolic Dependencies across
Colorectal Cancer Progression. Mol. Metab..

[2] La
Vecchia S., Sebastián C. (2020). Metabolic Pathways Regulating Colorectal
Cancer Initiation and Progression. Semin. Cell
Dev. Biol..

[3] Li Q., Geng S., Luo H., Wang W., Mo Y.-Q., Luo Q., Wang L., Song G.-B., Sheng J.-P., Xu B. (2024). Signaling
Pathways Involved in Colorectal Cancer: Pathogenesis and Targeted
Therapy. Signal Transduction and Targeted Therapy
2024 9:1.

[4] Singer M., Valerin J., Zhang Z., Zhang Z., Dayyani F., Yaghmai V., Choi A., Imagawa D., Abi-Jaoudeh N. (2025). Promising
Cellular Immunotherapy for Colorectal Cancer Using Classical Dendritic
Cells and Natural Killer T Cells. Cells.

[5] Kjaer T. K., Moustsen-Helms I. R., Albieri V., Larsen S. B., Degett T. H., Tjønneland A., Johansen C., Kjaer S. K., Gogenur I., Dalton S. O. (2021). Risk of
Pharmacological or Hospital Treatment for Depression
in Patients with Colorectal Cancer–Associations with Pre-Cancer
Lifestyle, Comorbidity and Clinical Factors. Cancers (Basel)..

[6] Bardaweel S. K., Jaradat E., Hajjo R., AlJarrah H. (2025). Unraveling the Anticancer
Potential of SSRIs in Prostate Cancer by Combining Computational Systems
Biology and In Vitro Analyses. ACS Omega.

[7] Taler M., Gil-Ad I., Brener I., Hornfeld S. H., Weizman A. (2022). Complex Effects
of Sertraline and Citalopram on In Vitro Murine Breast Cancer Proliferation
and on In Vivo Progression and Anxiety Level. International Journal of Molecular Sciences 2022 Vol. 23, Page 2711.

[8] Dong F., He K., Zhang S., Song K., Jiang L., Hu L. P., Li Q., Zhang X. L., Zhang N., Li B. T., Zhu L. L., Li J., Feng M., Gao Y., Chen J., Hu X., Wang J., Jiang C., Wang C., Zhu H. H., Da L. T., Ji J., Zhang Z. G., Bao Z., Jiang S. H. (2024). SSRI Antidepressant Citalopram Reverses the Warburg
Effect to Inhibit Hepatocellular Carcinoma by Directly Targeting GLUT1. Cell Rep..

[9] He L., Fu Y., Tian Y., Wang X., Zhou X., Ding R. B., Qi X., Bao J. (2023). Antidepressants as
Autophagy Modulators for Cancer
Therapy. Molecules.

[10] Ahmadian E., Eftekhari A., Babaei H., Nayebi A. M., Eghbal M. A. (2017). Anti-Cancer
Effects of Citalopram on Hepatocellular Carcinoma Cells Occur via
Cytochrome C Release and the Activation of NF-KB. Anticancer Agents Med. Chem..

[11] Dong F., He K., Zhang S., Song K., Jiang L., Hu L. P., Li Q., Zhang X. L., Zhang N., Li B. T., Zhu L. L., Li J., Feng M., Gao Y., Chen J., Hu X., Wang J., Jiang C., Wang C., Zhu H. H., Da L. T., Ji J., Zhang Z. G., Bao Z., Jiang S. H. (2024). SSRI Antidepressant Citalopram Reverses the Warburg
Effect to Inhibit Hepatocellular Carcinoma by Directly Targeting GLUT1. Cell Rep..

[12] Xia Z., Lundgren B., Bergstrand A., DePierre J. W., Nässberger L. (1999). Changes in
the Generation of Reactive Oxygen Species and in Mitochondrial Membrane
Potential during Apoptosis Induced by the Antidepressants Imipramine,
Clomipramine, and Citalopram and the Effects on These Changes by Bcl-2
and Bcl-X­(L). Biochem. Pharmacol..

[13] Dong F., Zhang S., Song K., Jiang L., Hu L.-P., Li Q., Zhang X.-L., Li J., Feng M., Cai Z.-W., Yao H.-F., Li R.-K., Li H., Chen J., Hu X., Wang J., Jiang C., Zhu H. H., Wang C., Da L.-T., Zhang Z.-G., Bao Z., Wang X., Jiang S.-H. (2025). Citalopram Exhibits Immune-Dependent Anti-Tumor Effects
by Modulating C5aR1+ TAMs and CD8+ T Cells. elife.

[14] Nenkov M., Ma Y., Gaßler N., Chen Y. (2021). Metabolic Reprogramming of Colorectal
Cancer Cells and the Microenvironment: Implication for Therapy. Int. J. Mol. Sci..

[15] Yang J., Shay C., Saba N. F., Teng Y. (2024). Cancer Metabolism and
Carcinogenesis. Experimental Hematology &
Oncology 2024 13:1.

[16] Kaur B., Sohrabi Y., Achreja A., Lisanti M. P., Martinez-Outschoorn U. E. (2023). Editorial:
Hallmark of Cancer: Reprogramming of Cellular Metabolism. Front. Oncol..

[17] Liu L., Wang H., Chen X., Zhang Y., Zhang H., Xie P. (2023). Gut Microbiota and Its Metabolites in Depression: From Pathogenesis
to Treatment. EBioMedicine.

[18] Jiang Y., Qu Y., Shi L., Ou M., Du Z., Zhou Z., Zhou H., Zhu H. (2024). The Role of
Gut Microbiota and Metabolomic
Pathways in Modulating the Efficacy of SSRIs for Major Depressive
Disorder. Transl. Psychiatry.

[19] Xu F., Xie Q., Kuang W., Dong Z. (2023). Interactions Between
Antidepressants
and Intestinal Microbiota. Neurotherapeutics.

[20] Sjöstedt P., Enander J., Isung J. (2021). Serotonin
Reuptake Inhibitors and
the Gut Microbiome: Significance of the Gut Microbiome in Relation
to Mechanism of Action, Treatment Response, Side Effects, and Tachyphylaxis. Front. Psychiatry.

[21] Brozek-Pluska B., Beton K. (2021). Oxidative Stress Induced by: T BHP
in Human Normal Colon Cells by
Label Free Raman Spectroscopy and Imaging. The Protective Role of
Natural Antioxidants in the Form of β-Carotene. RSC Adv..

[22] Beton K., Brożek-Płuska B. (2022). Biochemistry
and Nanomechanical Properties
of Human Colon Cells upon Simvastatin, Lovastatin, and Mevastatin
Supplementations: Raman Imaging and AFM Studies. J. Phys. Chem. B.

[23] Beton K., Wysocki P., Brozek-Pluska B. (2022). Mevastatin
in Colon Cancer by Spectroscopic
and Microscopic Methods – Raman Imaging and AFM Studies. Spectrochim. Acta A Mol. Biomol. Spectrosc..

[24] Collins T. J. (2007). ImageJ
for Microscopy. Biotechniques.

[25] ImageJ. https://imagej.net/ij/images/ (accessed 2020–06–14).

[26] Altman F. P. (1976). Tetrazolium
Salts and Formazans. Prog. Histochem. Cytochem..

[27] Berridge M. V., Herst P. M., Tan A. S. (2005). Tetrazolium
Dyes as Tools in Cell
Biology: New Insights into Their Cellular Reduction. Biotechnol. Annu. Rev..

[28] Marshall N. J., Goodwin C. J., Holt S. J. (1995). A Critical Assessment
of the Use
of Microculture Tetrazolium Assays to Measure Cell Growth and Function. Growth Regul..

[29] Scudiero D. A., Shoemaker R. H., Paull K. D., Monks A., Tierney S., Nofziger T. H., Currens M. J., Seniff D., Boyd M. R. (1988). Evaluation
of a Soluble Tetrazolium/Formazan Assay for Cell Growth and Drug Sensitivity
in Culture Using Human and Other Tumor Cell Lines. Cancer Res..

[30] Pang B., Wu H. (2025). Metabolic Reprogramming in Colorectal Cancer: A Review of Aerobic
Glycolysis and Its Therapeutic Implications for Targeted Treatment
Strategies. Cell Death Discovery.

[31] Avendaño-Félix M., Aguilar-Medina M., Bermudez M., Lizárraga-Verdugo E., López-Camarillo C., Ramos-Payán R. (2020). Refocusing
the Use of Psychiatric Drugs for Treatment of Gastrointestinal Cancers. Front. Oncol..

[32] Schiliro C., Firestein B. L. (2021). Mechanisms of Metabolic Reprogramming in Cancer Cells
Supporting Enhanced Growth and Proliferation. Cells.

[33] Karmakar S., Lal G. (2021). Role of Serotonin Receptor Signaling in Cancer Cells and Anti-Tumor
Immunity. Theranostics.

[34] Beton-Mysur K., Brożek-Płuska B. (2025). Exploring
the Impact of Citalopram
on Human Colon Cells: Insights into Antidepressant Action Beyond the
Brain. Spectrochim. Acta A Mol. Biomol. Spectrosc..

[35] Zheng Y., Chang X., Huang Y., He D. (2023). The Application of
Antidepressant Drugs in Cancer Treatment. Biomedicine
& Pharmacotherapy.

[36] Ahmadian E., Eftekhari A., Babaei H., Nayebi A. M., Eghbal M. A. (2017). Anti-Cancer
Effects of Citalopram on Hepatocellular Carcinoma Cells Occur via
Cytochrome C Release and the Activation of NF-KB. Anticancer Agents Med. Chem..

[37] Chen L., Huang S., Wu X., He W., Song M. (2024). Serotonin
Signalling in Cancer: Emerging Mechanisms and Therapeutic Opportunities. Clin. Transl. Med..

[38] Duan S., Fu Y., Dong S., Ma Y., Meng H., Guo R., Chen J., Liu Y., Li Y. (2022). Psychoactive Drugs
Citalopram and Mirtazapine Caused Oxidative Stress and Damage of Feeding
Behavior in Daphnia Magna. Ecotoxicol. Environ.
Saf..

[39] Yu H., Qu T., Yang J., Dai Q. (2023). Serotonin Acts through YAP to Promote
Cell Proliferation: Mechanism and Implication in Colorectal Cancer
Progression. Cell Communication and Signaling.

[40] Musa M., Ouaret D., Bodmer W. F. (2020). In Vitro
Analyses of Interactions
between Colonic Myofibroblasts and Colorectal Cancer Cells for Anticancer
Study. Anticancer Res..

[41] Micalet A., Upadhyay A., Javanmardi Y., de Brito C. G., Moeendarbary E., Cheema U. (2024). Patient-Specific Colorectal-Cancer-Associated
Fibroblasts
Modulate Tumor Microenvironment Mechanics. iScience.

[42] Henriksson M. L., Edin S., Dahlin A. M., Oldenborg P. A., Öberg Å., Van Guelpen B., Rutegård J., Stenling R., Palmqvist R. (2011). Colorectal Cancer Cells Activate
Adjacent Fibroblasts Resulting in FGF1/FGFR3 Signaling and Increased
Invasion. Am. J. Pathol..

[43] Ballou Y., Rivas A., Belmont A., Patel L., Amaya C., Lipson S., Khayou T., Dickerson E., Nahleh Z., Bryan B. (2018). 5-HT Serotonin Receptors
Modulate
Mitogenic Signaling and Impact Tumor Cell Viability. Mol. Clin. Oncol..

[44] Oufkir T., Arseneault M., Sanderson J. T., Vaillancourt C. (2010). The 5-HT2A
Serotonin Receptor Enhances Cell Viability, Affects Cell Cycle Progression
and Activates MEK–ERK1/2 and JAK2–STAT3 Signalling Pathways
in Human Choriocarcinoma Cell Lines. Placenta.

[45] Karmakar S., Lal G. (2021). Role of Serotonin Receptor Signaling in Cancer Cells and Anti-Tumor
Immunity. Theranostics.

[46] Pellarin I., Dall’Acqua A., Favero A., Segatto I., Rossi V., Crestan N., Karimbayli J., Belletti B., Baldassarre G. (2025). Cyclin-Dependent
Protein Kinases and Cell Cycle Regulation in Biology and Disease. Signal Transduct. Target. Ther..

[47] Ding L., Cao J., Lin W., Chen H., Xiong X., Ao H., Yu M., Lin J., Cui Q. (2020). The Roles of Cyclin-Dependent Kinases
in Cell-Cycle Progression and Therapeutic Strategies in Human Breast
Cancer. Int. J. Mol. Sci..

[48] Chesnokova V., Pechnick R. N. (2008). Antidepressants
and Cdk Inhibitors: Releasing the Brake
on Neurogenesis?. Cell Cycle.

[49] Ghafouri-Fard S., Khoshbakht T., Hussen B. M., Dong P., Gassler N., Taheri M., Baniahmad A., Dilmaghani N. A. (2022). A Review
on the Role of Cyclin Dependent Kinases in Cancers. Cancer Cell Int..

[50] Afzal S., Abdul Manap A. S., Attiq A., Albokhadaim I., Kandeel M., Alhojaily S. M. (2023). From Imbalance to Impairment: The
Central Role of Reactive Oxygen Species in Oxidative Stress-Induced
Disorders and Therapeutic Exploration. Front.
Pharmacol..

[51] Zheng Y., Chang X., Huang Y., He D. (2023). The Application of
Antidepressant Drugs in Cancer Treatment. Biomedicine
& Pharmacotherapy.

[52] Xia Z., Bergstrand A., Depierre J. W., Nässberger L. (1999). The Antidepressants
Imipramine, Clomipramine, and Citalopram Induce Apoptosis in Human
Acute Myeloid Leukemia HL-60 Cells via Caspase-3 Activation. J. Biochem. Mol. Toxicol..

[53] Salama M., Elamin A., Youssif M., Mattar N. A. (2025). Induction of DNA
Damage and Growth Arrest by Citalopram in Breast Cancer Cells Mediated
via Activation of Gadd45a and Apoptotic Genes. Ultrastruct. Pathol..

[54] Salama M., Ali A., Ibrahim F. A. R., Elabd S. (2024). Citalopram, an Antipsychotic
Agent, Induces G1/G0 Phase Cell Cycle Arrest and Promotes Apoptosis
in Human Laryngeal Carcinoma HEP-2 Cells. Medical
Oncology.

[55] Brandl N., Seitz R., Sendtner N., Müller M., Gülow K. (2025). Living on the Edge: ROS Homeostasis
in Cancer Cells
and Its Potential as a Therapeutic Target. Antioxidants.

[56] Aboelella N. S., Brandle C., Kim T., Ding Z. C., Zhou G. (2021). Oxidative
Stress in the Tumor Microenvironment and Its Relevance to Cancer Immunotherapy. Cancers (Basel)..

[57] Reddy A. P., Sawant N., Morton H., Kshirsagar S., Bunquin L. E., Yin X., Reddy P. H. (2021). Selective Serotonin
Reuptake Inhibitor Citalopram Ameliorates Cognitive Decline and Protects
against Amyloid Beta-Induced Mitochondrial Dynamics, Biogenesis, Autophagy,
Mitophagy and Synaptic Toxicities in a Mouse Model of Alzheimer’s
Disease. Hum. Mol. Genet..

[58] Xia Z., Lundgren B., Bergstrand A., DePierre J. W., Nässberger L. (1999). Changes in
the Generation of Reactive Oxygen Species and in Mitochondrial Membrane
Potential during Apoptosis Induced by the Antidepressants Imipramine,
Clomipramine, and Citalopram and the Effects on These Changes by Bcl-2
and Bcl-X­(L). Biochem. Pharmacol..

[59] Chen L., Huang S., Wu X., He W., Song M. (2024). Serotonin
Signalling in Cancer: Emerging Mechanisms and Therapeutic Opportunities. Clin. Transl. Med..

[60] Ahmadian E., Eftekhari A., Babaei H., Nayebi A. M., Eghbal M. A. (2017). Anti-Cancer
Effects of Citalopram on Hepatocellular Carcinoma Cells Occur via
Cytochrome C Release and the Activation of NF-KB. Anticancer Agents Med. Chem..

[61] Morse P. T., Arroum T., Wan J., Pham L., Vaishnav A., Bell J., Pavelich L., Malek M. H., Sanderson T. H., Edwards B. F. P., Hüttemann M. (2024). Phosphorylations
and Acetylations
of Cytochrome c Control Mitochondrial Respiration, Mitochondrial Membrane
Potential, Energy, ROS, and Apoptosis. Cells
2024, Vol. 13, Page 493.

[62] Koundouros N., Poulogiannis G. (2020). Reprogramming of Fatty Acid Metabolism in Cancer. Br. J. Cancer.

[63] Renne M. F., Hariri H. (2021). Lipid Droplet-Organelle
Contact Sites as Hubs for Fatty
Acid Metabolism, Trafficking, and Metabolic Channeling. Front. Cell Dev. Biol..

[64] Jutras-Carignan A., Guillemette T., Mounier C. (2023). From Endoplasmic Reticulum
to Nucleus:
The Fate of Cellular Fatty Acids. Cellular Lipid
in Health and Disease.

[65] Jacquemyn J., Cascalho A., Goodchild R. E. (2017). The Ins
and Outs of Endoplasmic Reticulum-controlled
Lipid Biosynthesis. EMBO Rep..

[66] Uchida Y. (2011). The Role of
Fatty Acid Elongation in Epidermal Structure and Function. Dermatoendocrinol..

[67] Smolková K., Gotvaldová K. (2025). Fatty Acid Trafficking Between Lipid Droplets and Mitochondria:
An Emerging Perspective. Int. J. Biol. Sci..

[68] Lu X., Li G., Liu Y., Luo G., Ding S., Zhang T., Li N., Geng Q. (2024). The Role of Fatty Acid Metabolism in Acute Lung Injury:
A Special Focus on Immunometabolism. Cell. Mol.
Life Sci..

[69] Phillips M. J., Voeltz G. K. (2016). Structure and Function
of ER Membrane Contact Sites
with Other Organelles. Nat. Rev. Mol. Cell Biol..

[70] Almeida C., Amaral M. D. (2020). A Central Role of
the Endoplasmic Reticulum in the
Cell Emerges from Its Functional Contact Sites with Multiple Organelles. Cell. Mol. Life Sci..

[71] English A. R., Voeltz G. K. (2013). Endoplasmic Reticulum
Structure and Interconnections
with Other Organelles. Cold Spring Harb. Perspect.
Biol..

[72] Zhang Y., Wu Y., Zhang M., Li Z., Liu B., Liu H., Hao J., Li X. (2023). Synergistic Mechanism
between the Endoplasmic Reticulum
and Mitochondria and Their Crosstalk with Other Organelles. Cell Death Discovery.

[73] Kuijpers M., Nguyen P. T., Haucke V. (2024). The Endoplasmic Reticulum
and Its
Contacts: Emerging Roles in Axon Development, Neurotransmission, and
Degeneration. Neuroscientist.

[74] Cohen S., Valm A. M., Lippincott-Schwartz J. (2018). Interacting
Organelles. Curr. Opin. Cell Biol..

[75] Wu H., Carvalho P., Voeltz G. K. (2018). Here, There,
and Everywhere: The
Importance of ER Membrane Contact Sites. Science.

[76] Szkalisity Á., Vanharanta L., Saito H., Vörös C., Li S., Isomäki A., Tomberg T., Strachan C., Belevich I., Jokitalo E., Ikonen E. (2025). Nuclear Envelope-Associated
Lipid Droplets Are Enriched in Cholesteryl Esters and Increase during
Inflammatory Signaling. EMBO J..

[77] Yao Y., Ding L., Huang X. (2020). Diverse Functions
of Lipids and Lipid
Metabolism in Development. Small Methods.

[78] Robbins A. L., Savage D. B. (2015). The Genetics of
Lipid Storage and Human Lipodystrophies. Trends
Mol. Med..

[79] Welte M. A., Gould A. P. (2017). Lipid Droplet Functions beyond Energy
Storage. Biochim. Biophys. Acta.

[80] Geltinger F., Schartel L., Wiederstein M., Tevini J., Aigner E., Felder T. K., Rinnerthaler M. (2020). Friend or
Foe: Lipid Droplets as
Organelles for Protein and Lipid Storage in Cellular Stress Response,
Aging and Disease. Molecules 2020, Vol. 25,
Page 5053.

[81] Ruart D., Riedinger J., Zitouni S., Bienvenu A., Bonazzi M., Martinez E. (2025). Bacterial Puppeteering: How the Stealth Bacterium Coxiella
Pulls the Cellular Strings. Pathogens.

[82] Chandra P., Banerjee S., Saha P., Chawla-Sarkar M., Patra U. (2022). Sneaking into the Viral Safe-Houses:
Implications of Host Components
in Regulating Integrity and Dynamics of Rotaviral Replication Factories. Front. Cell. Infect. Microbiol..

[83] Mukherjee R., Dikic I. (2022). Regulation of Host-Pathogen
Interactions via the Ubiquitin System. Annu.
Rev. Microbiol..

[84] Bosch M., Pol A. (2022). Eukaryotic Lipid Droplets:
Metabolic Hubs, and Immune First Responders. Trends in Endocrinology & Metabolism.

[85] Boucher D. M., Vijithakumar V., Ouimet M. (2021). Lipid Droplets as Regulators of Metabolism
and Immunity. Immunometabolism (United States).

[86] Movasaghi Z., Rehman S., Rehman I. U. (2007). Raman Spectroscopy
of Biological
Tissues. Appl. Spectrosc. Rev..

[87] Kong K., Kendall C., Stone N., Notingher I. (2015). Raman Spectroscopy
for Medical Diagnostics — From in-Vitro Biofluid Assays to
in-Vivo Cancer Detection. Adv. Drug Delivery
Rev..

[88] Shetty G., Kendall C., Shepherd N., Stone N., Barr H. (2006). Raman Spectroscopy:
Elucidation of Biochemical Changes in Carcinogenesis of Oesophagus. Br. J. Cancer.

[89] Stone N., Kendall C., Shepherd N., Crow P., Barr H. (2002). Near-Infrared
Raman Spectroscopy for the Classification of Epithelial Pre-Cancers
and Cancers. J. Raman Spectrosc..

[90] Stone N., Kendall C., Smith J., Crow P., Barr H. (2004). Raman Spectroscopy
for Identification of Epithelial Cancers. Faraday
Discuss..

[91] Crow P., Barrass B., Kendall C., Hart-Prieto M., Wright M., Persad R., Stone N. (2005). The Use of Raman Spectroscopy
to Differentiate between Different Prostatic Adenocarcinoma Cell Lines. British Journal of Cancer 2005 92:12.

[92] Nallala J., Jeynes C., Saunders S., Smart N., Lloyd G., Riley L., Salmon D., Stone N. (2020). Characterization of
Colorectal Mucus Using Infrared Spectroscopy: A Potential Target for
Bowel Cancer Screening and Diagnosis. Lab. Invest..

[93] Crow P., Stone N., Kendall C. A., Uff J. S., Farmer J. A. M., Barr H., Wright M. P. J. (2003). The Use of Raman Spectroscopy to
Identify and Grade Prostatic Adenocarcinoma in Vitro. Br. J. Cancer.

[94] Crow P., Stone N., Kendall C. A., Uff J. S., Farmer J. A. M., Barr H., Wright M. P. J. (2003). The
Use of Raman Spectroscopy to
Identify and Grade Prostatic Adenocarcinoma in Vitro. Br. J. Cancer.

[95] Orleanska J., Krol W., Majzner K. (2025). Assessing
Endothelial Cytotoxicity
Induced by Tyrosine Kinase Inhibitors: Insights from Raman and Fluorescence
Imaging. Analyst.

[96] Czamara K., Majzner K., Selmi A., Baranska M., Ozaki Y., Kaczor A. (2017). Unsaturated Lipid Bodies as a Hallmark of Inflammation
Studied by Raman 2D and 3D Microscopy. Scientific
Reports 2017 7:1.

[97] Majzner K., Tott S., Roussille L., Deckert V., Chlopicki S., Baranska M. (2018). Uptake of Fatty Acids by a Single Endothelial Cell
Investigated by Raman Spectroscopy Supported by AFM. In Analysis.

[98] Majzner K., Kochan K., Kachamakova-Trojanowska N., Maslak E., Chlopicki S., Baranska M. (2014). Raman Imaging Providing Insights
into Chemical Composition of Lipid Droplets of Different Size and
Origin: In Hepatocytes and Endothelium. Anal.
Chem..

[99] Szafraniec E., Majzner K., Farhane Z., Byrne H. J., Lukawska M., Oszczapowicz I., Chlopicki S., Baranska M. (2016). Spectroscopic Studies
of Anthracyclines: Structural Characterization and in Vitro Tracking. Spectrochim. Acta A Mol. Biomol. Spectrosc..

[100] Ravera F., Efeoglu E., Byrne H. J. (2021). Monitoring
Stem
Cell Differentiation Using Raman Microspectroscopy: Chondrogenic Differentiation,
towards Cartilage Formation. Analyst.

[101] Monaghan J. F., Byrne H. J., Lyng F. M., Meade A. D. (2024). Radiobiological
Applications of Vibrational Spectroscopy: A Review of Analyses of
Ionising Radiation Effects in Biology and Medicine. Radiation.

[102] Lyng F. M., Traynor D., Ramos I. R. M., Bonnier F., Byrne H. J. (2015). Raman Spectroscopy for Screening and Diagnosis of Cervical
Cancer. Anal. Bioanal. Chem..

[103] Paraskevaidi M., Matthew B. J., Holly B. J., Hugh B. J., Thulya C. P. V., Loren C., StJohn C., Peter G., Callum G., Sergei K. G., Kamila K., Maria K., Kássio L. M. G., Pierre M. H. L., Evangelos P., Savithri P., John A. A., Alexandra S., Marfran S., Josep S. S., Gunjan T., Michael W., Bayden W. (2021). Clinical Applications of Infrared and Raman Spectroscopy
in the Fields of Cancer and Infectious Diseases. Appl. Spectrosc. Rev..

[104] Guo S., Beleites C., Neugebauer U., Abalde-Cela S., Afseth N. K., Alsamad F., Anand S., Araujo-Andrade C., Aškrabić S., Avci E., Baia M., Baranska M., Baria E., Batista
De Carvalho L. A. E., De Bettignies P., Bonifacio A., Bonnier F., Brauchle E. M., Byrne H. J., Chourpa I., Cicchi R., Cuisinier F., Culha M., Dahms M., David C., Duponchel L., Duraipandian S., El-Mashtoly S. F., Ellis D. I., Eppe G., Falgayrac G., Gamulin O., Gardner B., Gardner P., Gerwert K., Giamarellos-Bourboulis E. J., Gizurarson S., Gnyba M., Goodacre R., Grysan P., Guntinas-Lichius O., Helgadottir H., Grošev V. M., Kendall C., Kiselev R., Kölbach M., Krafft C., Krishnamoorthy S., Kubryck P., Lendl B., Loza-Alvarez P., Lyng F. M., Machill S., Malherbe C., Marro M., Marques M. P. M., Matuszyk E., Morasso C. F., Moreau M., Muhamadali H., Mussi V., Notingher I., Pacia M. Z., Pavone F. S., Penel G., Petersen D., Piot O., Rau J. V., Richter M., Rybarczyk M. K., Salehi H., Schenke-Layland K., Schlücker S., Schosserer M., Schütze K., Sergo V., Sinjab F., Smulko J., Sockalingum G. D., Stiebing C., Stone N., Untereiner V., Vanna R., Wieland K., Popp J., Bocklitz T. (2020). Comparability of Raman Spectroscopic Configurations:
A Large Scale Cross-Laboratory Study. Anal.
Chem..

[105] Kulbacka J., Chwiłkowska A., Bar J., Poła A., Banaś T., Gamian A., Saczko J. (2010). Oxidative Alterations
Induced in Vitro by the Photodynamic Reaction in Doxorubicin-Sensitive
(LoVo) and -Resistant (LoVoDX) Colon Adenocarcinoma Cells. Exp. Biol. Med..

[106] Bassal M. A. (2023). The Interplay between Dysregulated
Metabolism and Epigenetics
in Cancer. Biomolecules.

[107] Amissah H. A., Likhomanova R., Opoku G., Amissah T. A., Balogi Z., Török Z., Vigh L., Combs S. E., Shevtsov M. (2025). Plasma Membrane Epichaperome–Lipid
Interface:
Regulating Dynamics and Trafficking. Cells.

[108] Wu H., Zhang Q., Cao Z., Cao H., Wu M., Fu M., Huang T., Han X., Chang X., Liu Z. (2025). Integrated
Spatial Omics of Metabolic Reprogramming and the Tumor Microenvironment
in Pancreatic Cancer. iScience.

[109] Kong K., Kendall C., Stone N., Notingher I. (2015). Raman Spectroscopy
for Medical Diagnostics - From in-Vitro Biofluid Assays to in-Vivo
Cancer Detection. Adv. Drug Delivery Rev..

[110] Entezar-Almahdi E., Mohammadi-Samani S., Tayebi L., Farjadian F. (2020). Recent Advances
in Designing 5-Fluorouracil Delivery Systems: A Stepping Stone in
the Safe Treatment of Colorectal Cancer. Int.
J. Nanomedicine.

[111] Campbell S. E., Stone W. L., Whaley S. G., Qui M., Krishnan K. (2003). Gamma (γ) Tocopherol Upregulates Peroxisome Proliferator
Activated Receptor (PPAR) Gamma (γ) Expression in SW 480 Human
Colon Cancer Cell Lines. BMC Cancer 2003 3:1.

[112] Orleanska J., Bik E., Baranska M., Majzner K. (2024). Mechanisms
of Mitotic Inhibition in Human Aorta Endothelial Cells: Molecular
and Morphological in Vitro Spectroscopic Studies. Spectrochim. Acta A Mol. Biomol. Spectrosc..

[113] Czamara K., Adamczyk A., Stojak M., Radwan B., Baranska M. (2021). Astaxanthin as a New Raman Probe
for Biosensing of
Specific Subcellular Lipidic Structures: Can We Detect Lipids in Cells
under Resonance Conditions?. Cell. Mol. Life
Sci..

[114] Yang T., Chettri A., Radwan B., Matuszyk E., Baranska M., Dietzek B. (2021). Monitoring Excited-State Relaxation
in a Molecular Marker in Live Cells–a Case Study on Astaxanthin. Chem. Commun..

[115] Radwan B., Adamczyk A., Tott S., Czamara K., Kaminska K., Matuszyk E., Baranska M. (2020). Labeled vs.
Label-Free
Raman Imaging of Lipids in Endothelial Cells of Various Origins. Molecules.

[116] Matuszyk E., Adamczyk A., Radwan B., Pieczara A., Szcześniak P., Mlynarski J., Kamińska K., Baranska M. (2021). Multiplex Raman Imaging of Organelles in Endothelial
Cells. Spectrochim. Acta A Mol. Biomol. Spectrosc..

[117] Leszczenko P., Nowakowska A. M., Jakubowska J., Pastorczak A., Zabczynska M., Mlynarski W., Baranska M., Ostrowska K., Majzner K. (2024). Raman Spectroscopy
Can Recognize the KMT2A Rearrangement as a Distinct Subtype of Leukemia. Spectrochim. Acta A Mol. Biomol. Spectrosc..

[118] Borek-Dorosz A., Pieczara A., Orleanska J., Brzozowski K., Tipping W., Graham D., Bik E., Kubrak A., Baranska M., Majzner K. (2024). Raman Microscopy Reveals
How Cell Inflammation Activates Glucose and Lipid Metabolism. Biochimica et Biophysica Acta (BBA) - Molecular Cell Research.

[119] Leszczenko P., Nowakowska A. M., Dawiec P., Czuja K., Jakubowska J., Zabczynska M., Pastorczak A., Ostrowska K., Tott S., Mlynarski W., Baranska M., Majzner K. (2024). Advancing
Triage of Acute Lymphoblastic
Leukaemia Subtypes Diagnosis: Label-Free Raman Spectroscopy for Precise
Single-Cell Phenotyping and Subtype Classification. Analyst.

[120] Leszczenko P., Nowakowska A. M., Dawiec P., Czuja K., Jakubowska J., Zabczynska M., Pastorczak A., Ostrowska K., Tott S., Mlynarski W., Baranska M., Majzner K. (2024). Advancing
Triage of Acute Lymphoblastic
Leukaemia Subtypes Diagnosis: Label-Free Raman Spectroscopy for Precise
Single-Cell Phenotyping and Subtype Classification. Analyst.

[121] Majzner K., Czamara K., Pacia M. Z., Dybas J., Bik E., Chrabaszcz K., Wiercigroch E., Dorosz A., Wislocka A., Marzec K. M., Malek K., Baranska M. (2020). Vibrational Imaging
of Proteins: Changes in the Tissues and Cells in the Lifestyle Disease
Studies. Vibrational Spectroscopy in Protein
Research: from Purified Proteins to Aggregates and Assemblies.

[122] Borek-Dorosz A., Nowakowska A. M., Leszczenko P., Adamczyk A., Pieczara A., Jakubowska J., Pastorczak A., Ostrowska K., Ząbczyńska M., Sowinski K., Gruszecki W. I., Baranska M., Marzec K. M., Majzner K. (2022). Raman-Based Spectrophenotyping of the Most Important
Cells of the Immune System. J. Adv. Res..

[123] Xiong B., Xie Z., Song F., Chen H., Wang X., Jin Z., Han T., Li Y., Zhang D. (2022). DDR1 Promotes LoVo Cell Proliferation by Regulatingenergy
Metabolism:
Effect of DDR1 on CRC Cell Metabolic Reprogramming. Acta Biochim. Biophys. Sin. (Shanghai)..

[124] Cotte A. K., Aires V., Fredon M., Limagne E., Derangère V., Thibaudin M., Humblin E., Scagliarini A., De Barros J. P. P., Hillon P., Ghiringhelli F., Delmas D. (2018). Lysophosphatidylcholine
Acyltransferase 2-Mediated
Lipid Droplet Production Supports Colorectal Cancer Chemoresistance. Nat. Commun..

[125] Lee J., Ridgway N. D. (2018). Phosphatidylcholine Synthesis Regulates
Triglyceride
Storage and Chylomicron Secretion by Caco2 Cells. J. Lipid Res..

[126] McPhee M., Lee J., Salsman J., Pinelli M., Di Cara F., Rosen K., Dellaire G., Ridgway N. D. (2024). Nuclear
Lipid Droplets in Caco2 Cells Originate from Nascent Precursors and
in Situ at the Nuclear Envelope. J. Lipid Res..

[127] Verjan Garcia N., Hong K. U., Matoba N. (2023). The Unfolded
Protein
Response and Its Implications for Novel Therapeutic Strategies in
Inflammatory Bowel Disease. Biomedicines.

[128] Lee J. S., Zheng Z., Mendez R., Ha S. W., Xie Y., Zhang K. (2012). Pharmacologic ER Stress
Induces Non-Alcoholic Steatohepatitis
in an Animal Model. Toxicol. Lett..

[129] Fader Kaiser C. M., Romano P. S., Vanrell M. C., Pocognoni C. A., Jacob J., Caruso B., Delgui L. R. (2022). Biogenesis
and Breakdown
of Lipid Droplets in Pathological Conditions. Front. Cell Dev. Biol..

[130] Geltinger F., Schartel L., Wiederstein M., Tevini J., Aigner E., Felder T. K., Rinnerthaler M. (2020). Friend or
Foe: Lipid Droplets as Organelles for Protein and Lipid Storage in
Cellular Stress Response, Aging and Disease. Molecules 2020, Vol. 25, Page 5053.

[131] Doultsinos D., Avril T., Lhomond S., Dejeans N., Guédat P., Chevet E. (2017). Control of the Unfolded
Protein Response
in Health and Disease. SLAS Discovery.

[132] Hetz C., Chevet E., Oakes S. A. (2015). Proteostasis Control
by the Unfolded Protein Response. Nat. Cell
Biol..

[133] Koh B., Jeon H., Kim D., Kang D., Kim K. R. (2018). Effect
of Fibroblast Co-Culture on the Proliferation, Viability and Drug
Response of Colon Cancer Cells. Oncol. Lett..

[134] Horman S. R., To J., Lamb J., Zoll J. H., Leonetti N., Tu B., Moran R., Newlin R., Walker J. R., Orth A. P. (2017). Functional Profiling
of Microtumors
to Identify Cancer Associated Fibroblast-Derived Drug Targets. Oncotarget.

[135] Deng T., Du B., Xi X. (2023). [Colorectal Cancer
Cells Induce the Formation of Cancer-Associated Fibroblasts by Activating
the ERK Signaling Pathway in Fibroblasts]. J.
Southern Med. Univ..

[136] Prados J., Melguizo C., Ortiz R., Perazzoli G., Cabeza L., Álvarez P. J., Rodriguez-Serrano F., Aranega A. (2013). Colon Cancer Therapy: Recent Developments
in Nanomedicine
to Improve the Efficacy of Conventional Chemotherapeutic Drugs. Anticancer Agents Med. Chem..

[137] Tomecka P., Kunachowicz D., Górczyńska J., Gebuza M., Kuźnicki J., Skinderowicz K., Choromańska A. (2024). Factors Determining Epithelial-Mesenchymal
Transition
in Cancer Progression. Int. J. Mol. Sci..

[138] Chen Z., Behrendt R., Wild L., Schlee M., Bode C. (2025). Cytosolic Nucleic Acid Sensing as
Driver of Critical Illness: Mechanisms
and Advances in Therapy. Signal Transduction
and Targeted Therapy 2025 10:1.

[139] Lu L. (2006). Stress-Induced Corneal Epithelial
Apoptosis Mediated by K+ Channel
Activation. Prog. Retin. Eye Res..

[140] Wang W., Ma J., Jia W., Xu G. (2011). Periostin:
A Putative Mediator Involved in Tumour Resistance to Anti-Angiogenic
Therapy?. Cell Biol. Int..

[141] Liu M., Wang Y., Zhang Y., Hu D., Tang L., Zhou B., Yang L. (2025). Landscape of Small
Nucleic Acid Therapeutics:
Moving from the Bench to the Clinic as next-Generation Medicines. Signal Transduction and Targeted Therapy 2025 10:1.

[142] McCreery C. V., Alessi D., Mollo K., Fasano A., Zomorrodi A. R. (2025). Investigating
Intestinal Epithelium Metabolic Dysfunction
in Celiac Disease Using Personalized Genome-Scale Models. BMC Medicine 2025 23:1.

[143] Luo R., Liu J., Wang T., Zhao W., Wang Y., Wen J., Wang H., Ding S., Zhou X. (2025). The Landscape of Malignant
Transition: Unraveling Cancer Cell-of-Origin and Heterogeneous Tissue
Microenvironment. Cancer Lett..

[144] Bartz R., Li W. H., Venables B., Zehmer J. K., Roth M. R., Welti R., Anderson R. G. W., Liu P., Chapman K. D. (2007). Lipidomics Reveals That Adiposomes
Store Ether Lipids
and Mediate Phospholipid Traffic. J. Lipid Res..

[145] Feuerer N., Marzi J., Brauchle E. M., Carvajal
Berrio D. A., Billing F., Weiss M., Jakobi M., Schneiderhan-Marra N., Shipp C., Schenke-Layland K. (2021). Lipidome Profiling
with Raman Microspectroscopy Identifies Macrophage Response to Surface
Topographies of Implant Materials. Proc. Natl.
Acad. Sci. U. S. A..

[146] Wang B., Tontonoz P. (2018). Liver X Receptors in
Lipid Signalling
and Membrane Homeostasis. Nat. Rev. Endocrinol..

[147] Lund E. G., Kerr T. A., Sakai J., Li W. P., Russell D. W. (1998). CDNA Cloning
of Mouse and Human Cholesterol 25-Hydroxylases,
Polytopic Membrane Proteins That Synthesize a Potent Oxysterol Regulator
of Lipid Metabolism. J. Biol. Chem..

[148] Ammendolia D. A., Bement W. M., Brumell J. H. (2021). Plasma Membrane
Integrity: Implications for Health and Disease. BMC Biology 2021 19:1.

[149] Zhu T., Jiang Z., Ma Y., Hu Y. (2016). Preservation of Supported
Lipid Membrane Integrity from Thermal Disruption: Osmotic Effect. ACS Appl. Mater. Interfaces.

[150] Dias C., Nylandsted J. (2021). Plasma Membrane
Integrity in Health
and Disease: Significance and Therapeutic Potential. Cell Discovery 2020 7:1.

[151] Horn A., Jaiswal J. K. (2019). Structural and Signaling
Role of
Lipids in Plasma Membrane Repair. Curr. Top.
Membr..

[152] Sadžak A., Mravljak J., Maltar-Strmečki N., Arsov Z., Baranović G., Erceg I., Kriechbaum M., Strasser V., Přibyl J., Šegota S. (2020). The Structural
Integrity of the Model Lipid Membrane during Induced Lipid Peroxidation:
The Role of Flavonols in the Inhibition of Lipid Peroxidation. Antioxidants.

[153] Louie S. M., Roberts L. S., Mulvihill M. M., Luo K., Nomura D. K. (2013). Cancer Cells Incorporate and Remodel Exogenous Palmitate
into Structural and Oncogenic Signaling Lipids. Biochimica et Biophysica Acta (BBA) - Molecular and Cell Biology
of Lipids.

[154] Feng T., Zhang H., Zhou Y., Zhu Y., Shi S., Li K., Lin P., Chen J. (2024). Roles of Posttranslational
Modifications in Lipid Metabolism and Cancer Progression. Biomarker Research 2024 12:1.

[155] Simeone P., Tacconi S., Longo S., Lanuti P., Bravaccini S., Pirini F., Ravaioli S., Dini L., Giudetti A. M. (2021). Expanding Roles of De Novo Lipogenesis
in Breast Cancer. International Journal of Environmental
Research and Public
Health 2021, Vol. 18, Page 3575.

[156] Fernández L. P., Gómez de Cedrón M., Ramírez de Molina A. (2020). Alterations of Lipid Metabolism in
Cancer: Implications in Prognosis and Treatment. Front. Oncol..

[157] Germain N., Dhayer M., Boileau M., Fovez Q., Kluza J., Marchetti P. (2020). Lipid Metabolism and Resistance to
Anticancer Treatment. Biology 2020, Vol. 9,
Page 474.

[158] Delmas D., Mialhe A., Cotte A. K., Connat J. L., Bouyer F., Hermetet F., Aires V. (2025). Lipid Metabolism in
Cancer: Exploring Phospholipids as Potential Biomarkers. Biomedicine and Pharmacotherapy.

[159] Zheng Y., Chang X., Huang Y., He D. (2023). The Application
of Antidepressant Drugs in Cancer Treatment. Biomedicine & Pharmacotherapy.

[160] Abdel-Razaq W., Kendall D. A., Bates T. E. (2011). The Effects
of Antidepressants
on Mitochondrial Function in a Model Cell System and Isolated Mitochondria. Neurochemical Research 2010 36:2.

[161] He L., Fu Y., Tian Y., Wang X., Zhou X., Ding R. B., Qi X., Bao J. (2023). Antidepressants as
Autophagy Modulators for Cancer Therapy. Molecules.

[162] Alberts, B. ; Johnson, A. ; Lewis, J. ; Raff, M. ; Roberts, K. ; Walter, P. The Endoplasmic Reticulum. In Molecular Biology of the Cell, 4th ed.; Garland Science: 2002.

[163] Ventura R., Hernández-Alvarez M. I., Ventura R., Hernández-Alvarez M. I. (2022). Endoplasmic Reticulum: A Hub in Lipid
Homeostasis. Updates on Endoplasmic Reticulum.

[164] Jin Y., Tan Y., Wu J., Ren Z. (2023). Lipid Droplets: A Cellular
Organelle Vital in Cancer Cells. Cell Death
Discovery.

[165] Safi R., Sánchez-Álvarez M., Bosch M., Demangel C., Parton R. G., Pol A. (2023). Defensive-Lipid
Droplets: Cellular Organelles Designed for Antimicrobial Immunity. Immunol. Rev..

