# Supplementary material for: Tracking Metabolic Responses to Citalopram in Colon Cells with Raman Spectroscopy
Source: Anal Chem. 2026 Feb 6;98(6):4480–98. doi: 10.1021/acs.analchem.5c03360 (PMC13298817; doi:10.1021/acs.analchem.5c03360)
Supplement: Supplementary file 1 [file ac5c03360_si_001.pdf]

Supporting Information

Tracking Metabolic Responses to Citalopram in Colon Cells with Raman Spectroscopy

Karolina Beton-Mysur[1*], Beata Brozek-Pluska[1*]

[1]*Lodz University of Technology, Faculty of Chemistry, Institute of Applied Radiation Chemistry, Laboratory of Laser Molecular Spectroscopy, Wroblewskiego 15, 93-590 Lodz, Poland*

*Correspondence: beata.brozek-pluska@p.lodz.pl; Tel.: +48 42 631 31 92
karolina.beton@p.lodz.pl; Tel.: +48 42 631 31 92

Table of contents

1. Pairwise three-dimensional PCA score plots (PC1–PC3) in the fingerprint region from all investigated cell lines…...**S3**

2. Discussion of the results…...**S4**

To determine the most important frequencies that differentiate normal and cancerous human colon cells, as well as cancer cells of varying aggressiveness, in the absence and presence of citalopram, pairwise PCA (Principal Component Analysis) was performed. Figure S1 shows the pairwise PCA results based on mean Raman spectra from CCD-18 Co, Caco-2, and LoVo cell lines, either untreated or treated with citalopram for 3 and 24 h for ER, including three-dimensional PCA score plots and biplot representations.

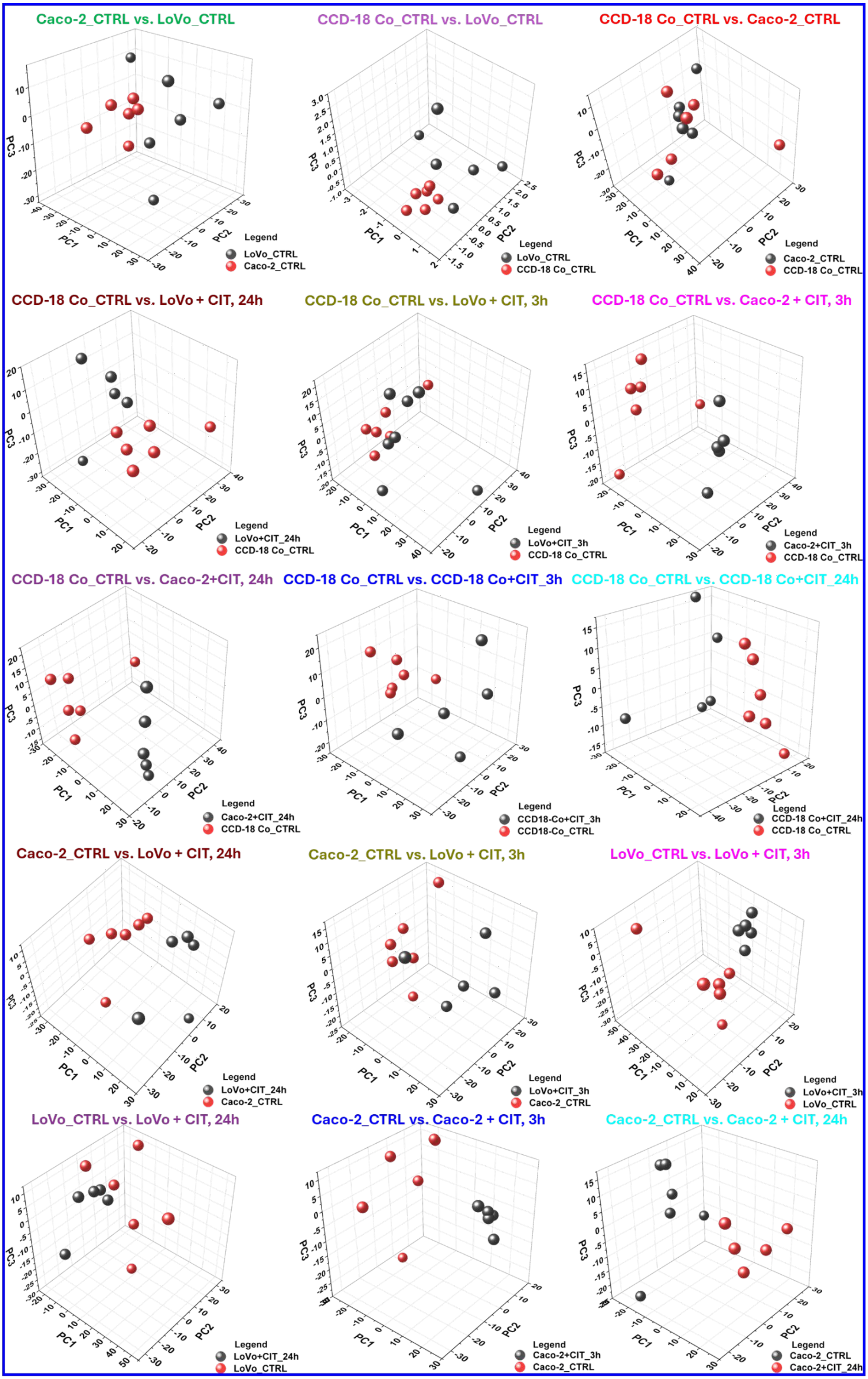
Caco-2_CTRL vs. LoVo_CTRL
Legend
LoVo_CTRL
Caco-2_CTRL
CCD-18 Co_CTRL vs. LoVo_CTRL
Legend
LoVo_CTRL
CCD-18 Co_CTRL
CCD-18 Co_CTRL vs. Caco-2_CTRL
Legend
Caco-2_CTRL
CCD-18 Co_CTRL
CCD-18 Co_CTRL vs. LoVo + CIT, 24h
Legend
LoVo+CIT_24h
CCD-18 Co_CTRL
CCD-18 Co_CTRL vs. LoVo + CIT, 3h
Legend
LoVo+CIT_3h
CCD-18 Co_CTRL
CCD-18 Co_CTRL vs. Caco-2 + CIT, 3h
Legend
Caco-2+CIT_3h
CCD-18 Co_CTRL
CCD-18 Co_CTRL vs. Caco-2+CIT, 24h
Legend
Caco-2+CIT_24h
CCD-18 Co_CTRL
CCD-18 Co_CTRL vs. CCD-18 Co+CIT_3h
Legend
CCD18-Co+CIT_3h
CCD18-Co_CTRL
CCD-18 Co_CTRL vs. CCD-18 Co+CIT_24h
Legend
CCD-18 Co+CIT_24h
CCD-18 Co_CTRL
Caco-2_CTRL vs. LoVo + CIT, 24h
Legend
LoVo+CIT_24h
Caco-2_CTRL
Caco-2_CTRL vs. LoVo + CIT, 3h
Legend
LoVo+CIT_3h
Caco-2_CTRL
LoVo_CTRL vs. LoVo + CIT, 3h
Legend
LoVo+CIT_3h
LoVo_CTRL
LoVo_CTRL vs. LoVo + CIT, 24h
Legend
LoVo+CIT_24h
LoVo_CTRL
Caco-2_CTRL vs. Caco-2 + CIT, 3h
Legend
Caco-2+CIT_3h
Caco-2_CTRL
Caco-2_CTRL vs. Caco-2 + CIT, 24h
Legend
Caco-2_CTRL
Caco-2+CIT_24h
PC1
PC2
PC3

Figure S1: *Pairwise three-dimensional PCA score plots (PC1–PC3) calculated from mean Raman spectra acquired in the fingerprint region from normal CCD-18 Co and cancerous Caco-2 and LoVo human colon cells, either untreated or supplemented with citalopram for 3 and 24 h. Each panel represents a pairwise comparison between cell types and/or treatment conditions. Raman spectra were collected using a 532 nm laser. For each experimental condition, spectra from five individual cells were collected and averaged before PCA. The fingerprint region covered the 600-1800 cm* $^{-1}$ *spectral range. Panel labels correspond to the comparisons indicated below each plot.*

Figure S1 demonstrates that the PCA algorithm effectively visualizes biochemical changes induced by drug exposure, as well as those occurring in non-supplemented cells. As illustrated in Figure S1, PC1 predominantly drives the differentiation of multiple control and citalopram-treated cell populations, differentiating (CCD18-Co_CTRL vs. LoVo+CIT, 24h), (CCD18-Co CTRL vs. LoVo+CIT, 3h), (CCD18-Co CTRL vs. Caco-2+CIT, 3h), (CCD18-Co CTRL vs. Caco-2+CIT, 24h), (CCD18-Co_CTRL vs. CCD18_Co+CIT_3h), (CCD-18-Co CTRL vs. CCD18 Co+CIT 24h), (Caco-2_CTRL vs. LoVo+CIT, 24h), (Caco-2_CTRL vs. Caco-2+CIT, 3h), and (Caco-2 CTRL vs. Caco-2+CIT, 24h). Simultaneously, PC2 contributes to the differentiation of (Caco-2 CTRL vs. LoVo+CIT, 3h), (LoVo CTRL vs. LoVo+CIT, 3h), and (LoVo CTRL vs. LoVo+CIT, 24h). In addition, PC3 provides supplementary discrimination within selected comparisons, particularly enhancing the separation between control and citalopram-treated samples within the same cell line, as well as contributing to the differentiation between Caco-2 and LoVo cell populations. Together, these results indicate that PC1 captures the dominant variance associated with both cell type and supplementation status, while PC2 reflects additional variance related to cell-line-specific and time-dependent responses to citalopram exposure. This indicates that PC3 captures more subtle variance related to finer biochemical differences and secondary cellular responses to citalopram supplementation.

Importantly, the PCA loadings define which specific Raman bands contribute most strongly to the variance captured by the principal components. Moreover, the figure confirms the mutual relationships between the corresponding PCA loadings and visually confirms the spectral differentiation between the experimental groups. On this basis, we selected the most prominent bands for subsequent Raman band ratio analysis to obtain biologically meaningful insights.
